# Combined Neurotoxic Effects of Commercial Formulations of Pyrethroid (Deltamethrin) and Neonicotinoid (Imidacloprid) Pesticides on Adult Zebrafish (*Danio rerio*): Behavioral, Molecular, and Histopathological Analysis

**DOI:** 10.3390/life15040538

**Published:** 2025-03-25

**Authors:** Adriana Petrovici, Gheorghe Savuța, Carla Lucini, Madalina-Andreea Robea, Carmen Solcan

**Affiliations:** 1 Faculty of Veterinary Medicine, “Ion Ionescu de la Brad” Iasi University of Life Sciences (IULS), 8 Mihail Sadoveanu Alley, 700489 Iasi, Romania; adriana.petrovici@iuls.ro (A.P.); gheorghe.savuta@iuls.ro (G.S.); 2 Department of Veterinary Medicine and Animal Production, University of Naples Federico II, 80137 Napoli, Italy; 3 Department of Biology, Doctoral School of Biology, Faculty of Biology, “Alexandru Ioan Cuza” University of Iasi, Bd. Carol I, 20 A, 700505 Iasi, Romania; madalina.robea11@gmail.com

**Keywords:** zebrafish brain, neonicotinoids, pyrethroids, neurotrophins, gene expression

## Abstract

The use of different commercial products that involve one or multiple active substances with specific targeted-pests control has become a widespread practice. Because of this, a severe range of significant consequences has been often reported. Among the most used pesticides worldwide are deltamethrin (DM) and imidacloprid (IMI). With a significative effect on the insect’s nervous system, DM acts on the voltage-gated sodium channels in nerve cell membranes, while IMI mimics the acetylcholine neurotransmitter by binding irreversibly to the nicotinic acetylcholine receptors. This study investigates the neurotoxic effects of sub-chronic exposure to commercial formulations of deltamethrin (DM) and imidacloprid (IMI) in adult zebrafish, both individually and in combination. The formulations used in this study contain additional ingredients commonly found in commercial pesticide products, which may contribute to overall toxicity. Fish were exposed to environmentally relevant concentrations of these pesticides for 21 days, individually or in combination. Behavioral, molecular, and histopathological analyses were conducted to assess the impact of these pesticides. Zebrafish exhibited dose-dependent behavioral alterations, particularly in the combined exposure groups, including increased erratic swimming and anxiety-like behavior. Gene expression analysis revealed significant changes in neurotrophic factors (*BDNF, NGF, ntf-3, ntf-4/5, ntf-6/7*) and their receptors (*ntrk1, ntrk2a, ntrk2b, ntrk3a, ntrk3b, ngfra, ngfrb*), indicating potential neurotoxic effects. Histopathological examination confirmed neuronal degeneration, gliosis, and vacuolization, with more severe impairments observed in pesticide mixture treatments. These findings highlight the neurotoxic potential of pesticide formulations in aquatic environments and emphasize the need for stricter regulations on pesticide mixtures and further research on pesticide interactions. Our findings emphasize that the combination of pesticides could trigger a synergistic effect by maximizing the toxicity of each compound. Thus, it is a well-known practice for pyrethroids and neonicotinoids to be used together in agriculture. Even so, its prevalence in agriculture and the need to investigate its actual impact on human health, biodiversity, and ecosystem mitigates the development of new strategies for assessing the risk and, at the same time, enhancing the effectiveness.

## 1. Introduction

Pesticides are essential in modern agriculture to protect crops [1] and increase productivity [2]. However, research is essential to comprehend its effects on food safety, the environment, and human health and create sustainable solutions. Pesticides are associated with significant risks to human health, including acute (poisoning) and chronic (cancer, endocrine disruption) toxicity [3]. Direct exposure mainly affects agricultural workers, and food residues pose a risk to consumers [4,5,6,7].

Pesticides affect biodiversity through negative impacts on non-target species, such as pollinators [8,9] and soil microorganisms [10], and contribute to water [11] and soil pollution [12]. For example, it has been estimated that pesticides kill approximately 72 million birds annually in the United States [13]. This impact on wildlife underlines the need to understand the individual effects of pesticides and how their combination can influence biodiversity. In addition, overuse leads to pest resistance, which complicates pest management and requires higher doses or more toxic substances [14]. Chemical interactions may lead to the formation of more toxic derivatives [15,16] or increased persistence of substances in soil and water [17,18].

Pesticide mixtures can lead to synergistic effects [19,20,21], where combinations of substances are more toxic than the sum of their individual effects. These interactions can intensify risks to human health and to organisms in the environment [22]. The combination of organophosphorus pesticides and carbamates can affect the central nervous system and impair the development of zebrafish embryos [23]. The effects of multiple pesticide exposures can accumulate dangerous doses over time, even if individual levels are considered safe. The US Environmental Protection Agency (EPA) analyzed multiple exposures to various organophosphates from various sources, such as food, drinking water, and residential uses [24]. The study integrated data on pesticide residue levels in food, concentrations in water, and used patterns in residential settings to estimate cumulative population exposure.

The results showed that, although individual exposures to each pesticide were below reference levels considered safe, cumulative exposure to multiple pesticides with similar toxic mechanisms could exceed safety thresholds, suggesting a potential risk to human health. These findings emphasized the importance of cumulative risk assessment in pesticide regulation and led to the implementation of measures to reduce the use of certain organophosphates and to promote safer agricultural practices [4,25,26,27].

In agriculture, crops are frequently treated with several pesticides simultaneously, exposing targeted and non-target organisms to various combinations. Detailed studies are needed to assess their effects, as traditional risk assessment methods usually focus on individual substances [28]. Current standards do not fully cover the risks associated with mixtures.

Mixtures can have different effects on different biological systems, including endocrine disruption [29,30,31] and chronic diseases [15,32,33,34,35]. Some combinations may amplify hormonal disruption, affecting reproduction and development. For example, triazole fungicide combinations have shown additive effects on progesterone production due to inhibition of cytochrome-P-450 (CYP) enzymes [36]. Long-term exposure to mixtures is associated with diseases such as cancer [37] or neurodegenerative conditions [38], which are more challenging to link to a single substance.

The zebrafish (*Danio rerio*) has become a favorite model in toxicological studies because of its unique characteristics, such as its genetic structure similar to that of higher vertebrates [39,40], the transparency of its larvae, and the ease of experimental manipulation [41,42,43,44,45]. This species is widely used to assess the harmful effects of environmental contaminants on the central nervous system (CNS) [46,47,48,49,50]. Among the most common chemical pollutants affecting aquatic environments are pesticides [51,52,53], especially neonicotinoids [54], and pyrethroids [55], two classes of compounds that have attracted attention because of their widespread use [9,11,12,56,57] and potential negative impacts on non-target organisms [34,58,59,60].

Neonicotinoids are a class of neuroactive, nicotine-derived insecticides that bind specifically to nicotinic acetylcholine receptors (nAChRs) [61], leading to hyperactivation of the nervous system and, ultimately, the death of target insects [62,63]. These compounds have gained popularity owing to their efficacy and ability to bind strongly to plant tissues, thereby providing long-lasting protection [64,65,66]. However, due to their persistence and solubility in water, neonicotinoids have become a source of concern for aquatic environments [67,68], with evidence that they can induce neurotoxic effects in other organisms [69,70,71], including zebrafish [72]. Long-term exposure to neonicotinoids has been associated with cognitive and behavioral dysfunction in fish, suggesting CNS impairment [73].

Pyrethroids, another important class of insecticides, are synthetic derivatives of pyrethrin, a natural substance found in chrysanthemum flowers [74,75]. Pyrethroids act by altering the function of neuronal sodium channels [76,77], which block the conduction of nerve impulses and the death of target insects. They are generally considered less toxic to mammals, but their toxicity to aquatic organisms is considerable [78,79], as fish and aquatic invertebrates are much more sensitive to these compounds [80]. Exposure of fish to pyrethroids can induce significant behavioral changes [47], such as reduced locomotor activity [81] and disruption of feeding and reproduction, indicative of CNS impairment [82].

This study investigated the effect of separate and combined exposure to neonicotinoids and pyrethroids on the central nervous system of adult zebrafish. The analysis of behavioral and neurochemical parameters aimed to elucidate the toxic effects of these pesticides, providing valuable information on the risks associated with the contamination of aquatic environments with such compounds. Understanding these effects is crucial for protecting aquatic biodiversity and developing strategies to regulate pesticide use to minimize ecosystem impacts.

We hypothesize that chronic exposure to deltamethrin (DM) and imidacloprid (IMI), both individually and in combination, induces neurotoxic effects in zebrafish, leading to behavioral, molecular, and histopathological alterations. Furthermore, we hypothesize that combined exposure will exacerbate toxicity due to possible synergistic interactions.

## 2. Materials and Methods

### 2.1. Fish Maintenance

Adult wild-type zebrafish (*Danio rerio*) were acquired in a total number of 240 from a nearby supplier in a 1:1 female-to-male sex ratio. Three weeks before the start of the experimental trial, the fish were housed in the facility. Daily water quality measurements of temperature, pH, salinity, conductivity, and ammonia were carried out to guarantee conditions peculiar to the environment (Table 1). The medium in the housing and experimental tanks was changed daily to prevent the buildup of metabolic waste products like ammonia. Along with water quality, the light cycle was supported by white LED bands (307.5 LUX) that were linked to an automated timer set to start at 8 a.m. and stop at 10 p.m. to maintain a circadian rhythm of 14:10 h (light:dark). Fish were fed ad libitum with TetraMin flakes (Herrenteich 78 49324 Melle, Germany) twice a day.

The EU Commission’s 2007 recommendation, the European Parliament’s Directive 2010/63/EU, and the Council’s 22 September 2010, standards for the housing, care, and protection of animals used for experiments and other scientific reasons were all followed in the maintenance and treatment of the animals, as well as those of Avdesh et al. and Aleström et al. [83,84].

The study was conducted with the approval of the Ethics Commission of the Faculty of Veterinary Medicine, University of Agricultural Sciences and Veterinary Medicine Iasi (Registration no. 749/4 July 2019).

### 2.2. Chemicals

The chemicals used in this study were bought from a designated shop as two different commercial products. The first product, DM, was designed as a liquid solution with the main component the active substance (100 g L^−1^ DM), and naphtha-solvent (aliphatic hydrocarbons). The second product, besides the active compound (100 g kg^−1^ IMI), the granular product also contained 10 g kg^−1^ benzenesulfonic acid, 10 g kg^−1^ 4-C10-14-alkyl derivatives, 10 g kg^−1^ calcium salts, 10 g kg^−1^ 2-methylpropan-1-ol, and 10 g kg^−1^ isobutanol.

The doses chosen for testing in the study on DM were 0.25 µg L^−1^, 0.5 µg L^−1^, and 1 µg L^−1^ DM (based on previous unpublished data). These were obtained by dissolving a certain amount of the prepared stock solution in the zebrafish medium.

The following concentrations were used for the IMI study: 12 µg L^−1^, 100 µg L^−1^, and 1000 µg L^−1^. The product was dissolved in warm water before administration.

In the mixed study, we combined DM with IMI in the following concentrations: 0.125 µg L^−1^ DM + 6 µg L^−1^ IMI; 0.25 µg L^−1^ DM + 50 µg L^−1^ IMI; and 0.5 µg L^−1^ DM + 500 µg L^−1^ IMI. For every study, we had a control group that did not receive any pesticide concentrations.

The single and combined doses were selected based on previous studies indicating environmentally relevant exposure levels. The selected doses reflect concentrations reported in agricultural runoff, ensuring ecological relevance [68,70,71,75,85,86,87,88,89,90] To maintain consistent pesticide concentrations, water was renewed every 24 h, and freshly prepared solutions of DM and IMI at the designated concentrations were used.

### 2.3. Experimental Design

Fish were randomly distributed from the housing tank to the experimental and control tanks, where they were allowed to get used to the new surroundings.

The present study was divided in three parts: first one was dedicated to the single exposure to DM (0.25 µg L^−1^, 0.5 µg L^−1^, and 1 µg L^−1^), the second one to IMI (12 µg L^−1^, 100 µg L^−1^, and 1000 µg L^−1^), and the third one to the combined exposure to DM and IMI (0.125 µg L^−1^ DM + 6 µg L^−1^ IMI, 0.25 µg L^−1^ DM + 50 µg L^−1^ IMI, and 0.5 µg L^−1^ DM + 500 µg L^−1^ IMI). Four tanks were set for each part of the study (three testing tanks and a control group tank). Each tank had a number of 10 animals. A stock solutions of DM (5 µg/mL DM stock solution) and IMI (10 mg/mL IMI stock solution) were prepared by dissolving the product in water. The exposure to each chemical was done by diluting a certain amount from the stock solution into fish’s medium. To maintain the dose of interest, the medium from the testing tanks was changed every 24 h. The study followed a 21 day period, with daily behavioral observations. In addition, the mortality rate was recorded as a consequence of exposure presence. At the end of exposure, fish were euthanized via submersion in ice-cold water for five minutes (until opercular movement ceased) and kept for further investigations (histological and molecular examination). Two additional replicates were included in the studies.

### 2.4. Behavioral Evaluation

Behavioral assessments were conducted to evaluate the neurotoxic effects of DM, IMI, and their combinations in adult zebrafish. Fish behavior was observed at predefined time points during the 21-day exposure period to identify and quantify neurotoxic manifestations.

#### 2.4.1. Behavioral Observation and Data Collection

Behavioral assessments were performed through direct observation by a trained observer in a quiet and controlled environment to minimize external stimuli that could influence zebrafish behavior. Each group was individually monitored in a glass observation tank (30 × 15 × 20 cm, filled with 5 L of clean water) under standard laboratory conditions (26 ± 1 °C, 14:10 h light-dark cycle). Behavioral observations were conducted at three fixed time points per day (before exposure, immediately after pesticide solution renewal, and 30 min after pesticide solution renewal) to account for potential behavioral variations. Each session lasted 5 min, during which behavioral abnormalities were recorded manually using a structured evaluation grid.

#### 2.4.2. Behavioral Parameters and Severity Scoring

Zebrafish were monitored for distinct neurotoxic behavioral indicators, with each behavior categorized based on predefined criteria. The presence or absence of each behavior was recorded for every individual, and a severity scoring system was applied to quantify behavioral impairment across experimental groups. The following parameters were assessed:

Hyperactivity: increased swimming speed, frequent darting, or sudden acceleration.

Erratic movements: characterized by abrupt, uncoordinated swimming patterns, including circling and jerky movements.

Thigmotaxis: time spent near the tank walls, indicative of heightened anxiety-like behavior.

Reduced exploration: decreased movement throughout the tank, often associated with stress or impaired motor function.

Immobility: prolonged stationary positioning with minimal fin or body movement.

Sickness-like behaviors: Manifestations such as floating near the water surface, sinking to the bottom, or loss of equilibrium.

Spiraling and motor incoordination: Uncontrolled circular swimming or inability to maintain a straight trajectory.

Floating and mouth-opening behavior: Unusual buoyancy issues and excessive mouth movements, potentially indicative of respiratory distress.

Each behavior was scored as present (✔) or absent (-) for every group, and the cumulative number of exhibited behaviors was used to determine the behavioral severity score for that group. A higher severity score indicated a greater degree of neurotoxic effects.

This structured manual observation approach and the binary score allowed for the systematic and quantitative assessment of behavioral impairments, providing insights into the neurotoxic impact of DM and IMI exposure.

### 2.5. Assessment of NTs and Their Receptors Gene Expression

To evaluate the expression levels of neurotrophins and their receptors in response to pesticide exposure, quantitative real-time PCR (qPCR) was performed following a rigorous workflow, ensuring high specificity, efficiency, and reproducibility.

#### 2.5.1. Sample Collection and RNA Extraction

Whole zebrafish brains were carefully dissected under a stereomicroscope (Nikon SMZ1270i, Nikon Corporation, Tokyo, Japan) and immediately stored in 1.5 mL microcentrifuge tubes at −80 °C until further processing. Total RNA was extracted using PeqGold TriFast (30-2010P, Peqlab, VWR International LLC, Radnor, PA, USA) following the manufacturer’s protocol. The integrity and purity of the extracted RNA were assessed spectrophotometrically, and only high-quality RNA samples (A260/A280 ratio between 1.8 and 2.0) were used for cDNA synthesis.

#### 2.5.2. cDNA Synthesis and Quantification

RNA was reverse-transcribed into complementary DNA (cDNA) using the SuperScript IV VILO cDNA Synthesis Kit (11766050, Invitrogen, Thermo Fisher Scientific, Waltham, MA, USA) in a 20-µL reaction volume. The synthesized cDNA was subsequently quantified using fluorometric analysis with a Qubit Fluorometer (Thermo Fisher Scientific, Waltham, MA, USA) and adjusted to a final working concentration of 20 ng/µL.

#### 2.5.3. Primer Validation and Standard Curve Generation

To ensure amplification specificity and efficiency, primers for each gene of interest (GOI) were validated before qPCR analysis. Primer pairs were assessed through a series of ten serial dilutions (1:10) of purified amplicons, obtained via initial PCR amplification. The amplicons were then confirmed for specificity using agarose gel electrophoresis, followed by purification with Agencourt Ampure XP beads (a63881, Beckman Coulter Inc.,Brea, CA, USA) and quantification via fluorometry (Qubit, Thermo Fisher Scientific, Waltham, MA, USA). These dilution series were subsequently used to generate standard curves in real-time PCR assays, allowing for the determination of amplification efficiency and dynamic range for each GOI.

#### 2.5.4. Quantitative Real-Time PCR (qPCR) Analysis

Real-time PCR reactions were conducted on a QuantStudio 12K Flex Real-Time PCR System (Thermo Fisher Scientific, Waltham, MA, USA) using SYBR-Green PCR Master Mix (4334973, Life Technologies LTD, Thermo Fisher Scientific, Waltham, MA, USA). The amplification protocol consisted of an initial denaturation step at 95 °C for 10 min, followed by 40 cycles of denaturation at 95 °C for 15 s and annealing/extension at 60–65 °C for 1 min. The primers used for qPCR reactions are listed in Table 2.

To ensure the accuracy and specificity of gene expression quantification, negative and positive controls were included in all qRT-PCR reactions. No-template controls (NTCs) were used to verify the absence of contamination or non-specific amplification by preparing reaction mixtures without cDNA templates. For positive controls, we utilized pre-generated standard curves obtained from serial dilutions (1:10) of purified and quantified PCR amplicons specific to each gene of interest. These standards served as reference points in every qRT-PCR run, allowing for precise quantification and validation of primer efficiency.

To enhance specificity and eliminate the potential influence of non-specific amplification or primer dimer formation, melting curve analysis was performed post-amplification, ensuring the detection of a single peak corresponding to the target amplicon.

#### 2.5.5. Data Normalization and Expression Calculation

Gene expression levels were normalized against elongation factor alpha (*ELFA*), which was selected as the most stable housekeeping gene based on preliminary stability assessments. The relative expression of each GOI was calculated using the following formula:

(GOI/GOR) × 100,

where GOI represents the quantified expression of the target gene, and GOR refers to the expression of the housekeeping gene (*ELFA*). This normalization approach ensures reliable quantification while minimizing variations due to differences in cDNA input or reaction efficiency.

All qPCR reactions were performed in technical duplicates, and data were analyzed using one-way ANOVA followed by Tukey’s post hoc test to compare gene expression across experimental groups. Results are presented as mean ± standard deviation (SD), with statistical significance set at *p* < 0.05.

### 2.6. Assessment of Histopathological Changes

To evaluate histopathological alterations in response to pesticide exposure, four zebrafish from each experimental group, including the control, were euthanized via immersion in ice-cold water following ethical euthanasia guidelines. The specimens were then fixed in 4% paraformaldehyde (PFA) at 4 °C for 24 h to preserve tissue morphology.

After fixation, the samples were decalcified in 0.35 M ethylenediaminetetraacetic acid (EDTA), pH 8, for five days, with the decalcification solution refreshed every 24 h to ensure complete calcium removal. The tissues were dehydrated through graded ethanol series, cleared in xylene, and embedded in paraffin. Serial 12-µm tissue sections were obtained using a rotary microtome and mounted onto poly-L-lysine-coated slides.

To assess structural integrity and detect potential neuronal damage, Luxol Fast Blue–Cresyl Violet (LFB-Cr. V) staining was performed. This enables the differentiation of gray and white matter regions and the visualization of neuronal and glial cell populations. Following staining, the sections were dehydrated, cleared, and coverslipped with DPX mounting medium for long-term preservation.

Histological analysis and high-resolution image acquisition were conducted using a Motic Easy Scan Pro 6 scanner system (Kowloon City, Kowloon, Hong Kong, Motic). The stained sections were examined for morphological alterations, including neuronal degeneration, gliosis, vacuolization, and structural disorganization. The histological features were qualitatively assessed and documented with digital imaging software.

### 2.7. Statistical Analysis

All behavioral, molecular, and mortality data were analyzed using one-way ANOVA and Tukey’s post hoc test to compare differences between experimental groups. Statistical significance was set at *p* < 0.05. Results are expressed as mean ± standard deviation (SD). Prior to statistical comparisons, all datasets were assessed for normality using the Shapiro–Wilk test, ensuring that parametric assumptions were met. In cases where normality was confirmed, one-way ANOVA was applied, followed by Tukey’s post hoc test to determine significant differences between exposure and control groups. Graphs and statistical analyses were generated using Python (version 3.10) [91] and the Matplotlib library version 3.6.3 [92].

## 3. Results

### 3.1. Zebrafish Behavior Assessment

Behavioral analysis revealed distinct patterns of neurotoxic effects following exposure to deltamethrin (DM), imidacloprid (IMI), and their combinations. The severity and frequency of behavioral alterations were strongly dose-dependent, with mixed-exposure groups exhibiting the most pronounced impairments. The severity scoring system used to quantify behavioral abnormalities indicated a progressive increase in neurotoxicity with higher pesticide concentrations, particularly in the combined exposure groups.

#### 3.1.1. Behavioral Severity Analysis

Statistical analysis revealed a significant dose-dependent increase in behavioral severity across experimental groups (*p* < 0.001, ANOVA, n = 10 per group). Pairwise comparisons showed that the 0.5 µg L^−1^ DM + 500 µg L^−1^ IMI group exhibited the highest severity score, significantly exceeding all other groups (*p* < 0.0001, Tukey post hoc test, n = 10 per group).

A clear dose-dependent trend was observed across the experimental groups, with the severity score increasing as pesticide concentrations increased. Control fish exhibited no behavioral abnormalities, while zebrafish exposed to low concentrations of DM (0.25 µg L^−1^) displayed minimal behavioral changes, resulting in a low severity score. Fish subjected to a moderate concentration of DM (0.5 µg L^−1^) exhibited a notable increase in behavioral abnormalities, including hyperactivity and erratic movements, which led to an intermediate severity score. Exposure to the highest concentration of DM (1 µg L^−1^) resulted in a dramatic increase in behavioral impairments, with fish exhibiting a broad range of neurotoxic behaviors such as motor incoordination, reduced feeding, and immobility, reflected in a near-maximal severity score of 14.

In contrast, exposure to IMI alone at concentrations of 12 µg L^−1^ and 100 µg L^−1^ did not induce significant behavioral changes, as indicated by a severity score of zero. However, zebrafish exposed to 1000 µg L^−1^ IMI exhibited moderate behavioral impairments, characterized by reduced exploration and sickness-like behaviors, resulting in a severity score of eight. These findings suggest that, at lower concentrations, IMI exerts negligible behavioral effects, while at higher concentrations, it induces mild to moderate neurotoxic manifestations.

The most severe behavioral disruptions were observed in zebrafish exposed to the combined pesticide treatments. The group exposed to 0.25 µg L^−1^ DM and 50 µg L^−1^ IMI displayed significantly increased hyperactivity, erratic swimming, and aggression, contributing to a severity score of 13, which was substantially higher than those observed in single-exposure groups at comparable concentrations. The highest combined exposure group, subjected to 0.5 µg L^−1^ DM and 500 µg L^−1^ IMI, exhibited the most extreme behavioral impairments, with all 15 assessed behaviors present, including spiraling, floating, and mouth-opening behavior. This group attained the highest possible severity score of 15, indicating a pronounced synergistic neurotoxic effect between DM and IMI.

**Figure 1:**
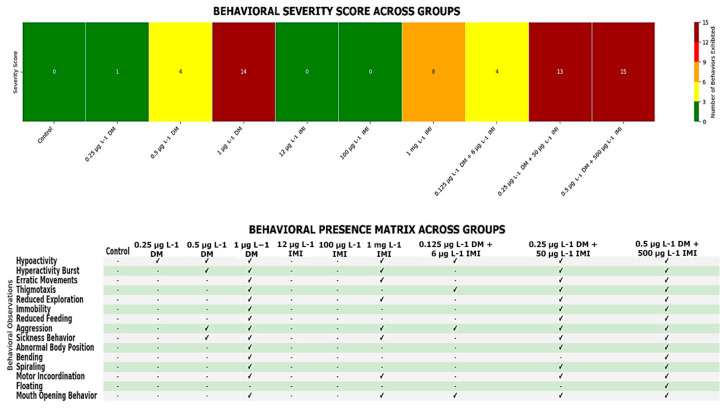
Behavioral severity across experimental groups—the top panel presents a heatmap illustrating the severity score for each experimental group, where colors represent the number of behavioral abnormalities observed (green = low, red = high). The bottom panel displays a behavioral presence matrix, indicating the presence (✔) or absence (-) of specific behavioral abnormalities across experimental groups. The severity score was calculated based on the total number of distinct behaviors exhibited within each group (n = 10 per group).

The presence of specific behaviors across experimental groups, as detailed in the behavioral presence table (Figure 1, bottom panel), supports the severity scoring results. Hyperactivity and hyperactivity bursts were observed in groups exposed to moderate and high concentrations of DM, as well as in mixed-exposure groups, suggesting that these pesticides induce nervous system overstimulation. Erratic movements and thigmotaxis, indicative of stress-induced anxiety responses, were common in groups exposed to high concentrations of DM and the DM-IMI mixtures. Reduced exploration and immobility increased progressively with pesticide concentration, particularly in high-dose DM and IMI groups, reflecting neurotoxic effects leading to motor impairment. Sickness-like behavior and reduced feeding were predominant in high-dose exposures, suggesting systemic toxicity affecting both neurological and metabolic functions. Aggression was notably present in the 0.25 µg L^−1^ DM + 50 µg L^−1^ IMI group, implying potential alterations in neurotransmission. More severe impairments such as abnormal body position, bending, spiraling, and motor incoordination were most pronounced in the 0.5 µg L^−1^ DM + 500 µg L^−1^ IMI group, consistent with severe neuromuscular dysfunction. Additionally, floating and mouth-opening behaviors were observed in highly affected groups, likely indicative of respiratory distress and loss of buoyancy control.

#### 3.1.2. Statistical Analysis of Behavioral Alterations

**Figure 2:**
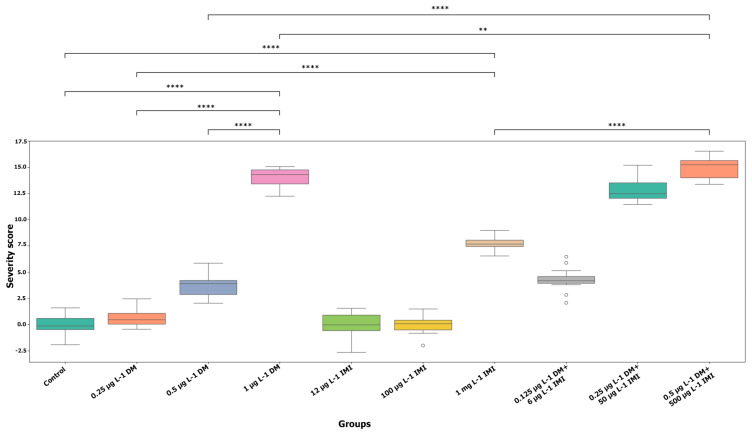
Behavioral severity across experimental groups with pairwise significance—box plot illustrating the distribution of behavioral severity scores across experimental groups. The y-axis represents the severity score, calculated as the total number of distinct behavioral abnormalities observed per group, while the x-axis denotes the different exposure conditions. Each box plot displays the median (horizontal line), interquartile range (box), and data variability (whiskers), with outliers represented as individual points. Statistical significance of pairwise comparisons between groups is indicated by horizontal brackets above the plot, with asterisks denoting significance levels (*p* < 0.01 (**), *p* < 0.0001 (****)).

The statistical analysis further confirmed the dose-dependent nature of pesticide-induced behavioral severity. Box plot analysis (Figure 2) revealed significant differences between experimental groups, with behavioral severity scores increasing systematically with pesticide concentration. Pairwise statistical comparisons demonstrated that zebrafish exposed to 1 µg L^−1^ DM and 1000 µg L^−1^ IMI exhibited significantly greater behavioral impairments than those exposed to lower concentrations, with *p*-values below 0.01. The most pronounced differences were observed in the mixed-exposure groups, where behavioral severity was significantly greater than in single-exposure groups. The combination of 0.5 µg L^−1^ DM and 500 µg L^−1^ IMI resulted in the most extreme neurotoxic manifestations, showing a statistically significant increase in severity compared to all other groups, with *p*-values below 0.0001.

The presence of highly significant differences among experimental groups was evident from the pairwise comparisons illustrated in Figure 2. Statistical significance was assessed using post hoc multiple comparison tests, with significance thresholds denoted by asterisks. Groups with moderate pesticide exposure exhibited significant behavioral alterations compared to control groups. In contrast, high-dose and mixed-exposure groups showed highly significant differences compared to their lower-dose counterparts. The severity scores in the 0.5 µg L^−1^ DM + 500 µg L^−1^ IMI group were statistically distinct from all other groups, indicating a strong synergistic toxic interaction between DM and IMI.

The statistical analysis underscores the strong dose-response relationship observed in the behavioral severity data. The results suggest that exposure to increasing concentrations of DM and IMI leads to progressively worsening neurotoxic effects. When combined, these pesticides exert a compounded impact that exceeds the sum of their individual toxicities. This cumulative and potentially synergistic effect highlights the heightened neurotoxicity associated with combined pesticide exposure.

### 3.2. Mortality Assessment

The evaluation of zebrafish mortality following exposure to DM, IMI, and their combinations provided critical insights into the lethal effects of these pesticides. Mortality rates were recorded at four time points (1, 7, 14, and 21 days post-exposure) to assess the progression of lethal effects over time. A linear regression analysis was performed for each exposure group to quantify mortality trends and determine the statistical significance of differences between groups.

#### Mortality Trends and Statistical Analysis

**Figure 3:**
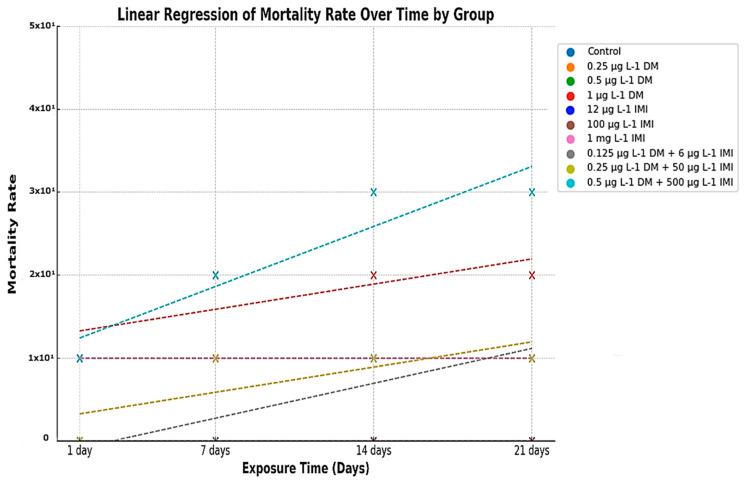
Linear regression of mortality rate over time across experimental groups. The *y*-axis represents the mortality rate (%) recorded at four-time points (1, 7, 14, and 21 days), while the *x*-axis denotes the exposure duration. Colored markers (X) indicate observed mortality data for each experimental group while corresponding dashed lines represent the linear regression fit for each condition. Statistical significance was assessed using ANOVA and post hoc comparisons to evaluate differences in mortality trends between exposure groups.

The analysis revealed a dose-dependent increase in mortality, with higher concentrations of DM and IMI leading to greater mortality rates over time (Figure 3). Control fish exhibited no mortality throughout the 21-day period, confirming that observed deaths in experimental groups were directly related to pesticide exposure.

Linear regression analysis demonstrated a significant increase in mortality rates over time in pesticide-exposed groups (*p* < 0.001, n = 10 per group). The highest mortality rate was 30% in the 0.5 µg L^−1^ DM + 500 µg L^−1^ IMI group, which was significantly higher than in single-exposure conditions (*p* < 0.0001, ANOVA and Tukey post hoc test, n = 10 per group).

In groups exposed to low concentrations of DM (0.25 µg L^−1^) and IMI (12 µg L^−1^), mortality remained below 5% at all time points, and statistical comparisons showed no significant differences from the control group (*p* > 0.05). Zebrafish subjected to moderate concentrations (0.5 µg L^−1^ DM, 100 µg L^−1^ IMI, and 0.125 µg L^−1^ DM + 6 µg L^−1^ IMI) exhibited slightly higher mortality rates, with values ranging between 5% and 10% by day 21. Despite this modest increase, statistical analysis confirmed that these groups had significantly higher mortality rates than the control (*p* < 0.05).

In contrast, higher pesticide concentrations (1 µg L^−1^ DM and 1000 µg L^−1^ IMI) resulted in substantial mortality, with rates reaching up to 20% by day 21. The highest mortality rates were observed in the combined pesticide exposure groups, particularly 0.5 µg L^−1^ DM + 500 µg L^−1^ IMI, where mortality reached 30% by day 21, significantly exceeding the rates observed in single-exposure groups (*p* < 0.001).

Linear regression analysis demonstrated a clear trend of increasing mortality over time in all pesticide-treated groups, with statistically significant positive slopes indicating a steady increase in mortality with prolonged exposure. The regression slopes were significantly steeper in high-dose and mixed-exposure groups, confirming that these conditions accelerated mortality progression compared to lower-dose treatments. Post hoc comparisons further revealed that the 0.5 µg L^−1^ DM + 500 µg L^−1^ IMI group exhibited significantly higher mortality rates than all other groups, including those receiving individual pesticide treatments (*p* < 0.0001).

### 3.3. NTs and Their Receptors Upregulated/Downregulated Gene Expression

The analysis of gene expression levels following exposure to various doses of DM, IMI, and their mixture reveals significant alterations in neurotrophin gene regulation. The bar plots summarize the results representing the relative mRNA levels of *BDNF*, *NGF*, *ntf-3*, and *ntf-4/5* across experimental groups and their receptors, respectively, TrkB (*ntrk2a* and *ntrk2b*) for *BDNF* and *ntf-4/5*, *ntrk1* for *NGF* and *ntf-6/7*, and *ntrk3a* and *ntrk3b* for *ntf-3*. The results for *ntf-6/7* showed no significant changes.

#### 3.3.1. *BDNF* and *TrkB* Expression

**Figure 4:**
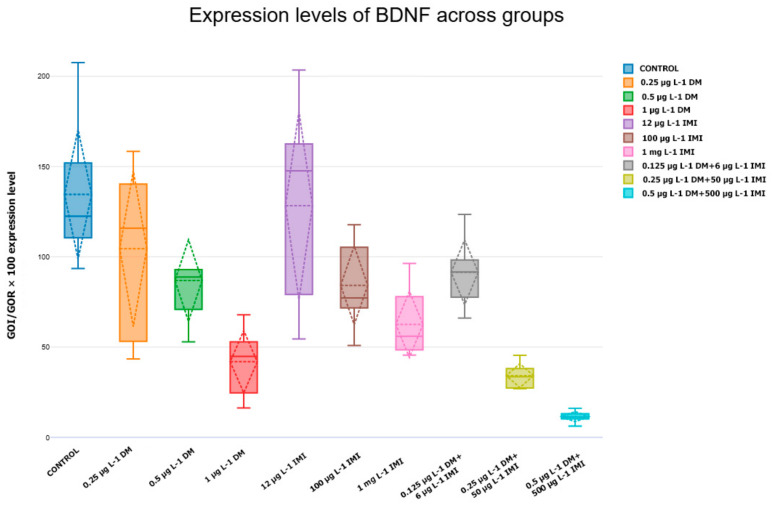
Expression levels of *BDNF* across experimental groups. The box plot represents the relative expression levels of *BDNF* in zebrafish (n = six per group) exposed to different concentrations of DM, IMI, and their combinations. The *y*-axis indicates the normalized gene expression (*GOI*/*GOR* × 100), while the *x*-axis represents the experimental groups. Each box plot displays the mean (horizontal discontinued line), median (horizontal continued line), interquartile range (box), and data variability (whiskers), with individual data points shown. The control group serves as the baseline reference.

The expression of *BDNF* varied significantly across experimental groups, showing both dose-dependent alterations and differential responses between single and combined pesticide exposures (Figure 4). The control group exhibited stable *BDNF* expression, serving as a reference for comparison.

Exposure to low concentrations of DM (0.25 µg L^−1^) resulted in a slight, non-significant increase in *BDNF* expression, which may suggest an initial compensatory neuroprotective response. However, at higher concentrations (0.5 µg L^−1^ and 1 µg L^−1^ DM), *BDNF* expression decreased significantly, with the 1 µg L^−1^ DM group showing the most pronounced reduction compared to the control (*p* < 0.01, Tukey post hoc, (n = six per group). This suggests a transition from an adaptive to a neurotoxic response with increasing DM exposure.

IMI exposure led to more variable expression patterns. The 12 µg L^−1^ and 100 µg L^−1^ IMI groups displayed heterogeneous responses, with some individuals showing increased expression while others exhibited downregulation. However, at 1000 µg L^−1^ IMI, *BDNF* expression was significantly reduced compared to the control (*p* < 0.05), indicating a neurotoxic effect at high IMI concentrations.

The most substantial reductions in *BDNF* expression were observed in the mixed-exposure groups. The 0.25 µg L^−1^ DM + 50 µg L^−1^ IMI condition showed a notable decline in *BDNF* levels compared to the control and respective single-exposure groups (*p* < 0.01). The 0.5 µg L^−1^ DM + 500 µg L^−1^ IMI group exhibited the lowest *BDNF* expression of all conditions, with highly significant differences compared to all other groups (*p* < 0.0001). These results confirm that co-exposure to DM and IMI has a synergistic neurotoxic effect, further suppressing *BDNF* expression beyond what is observed with individual pesticide exposure.

**Figure 5:**
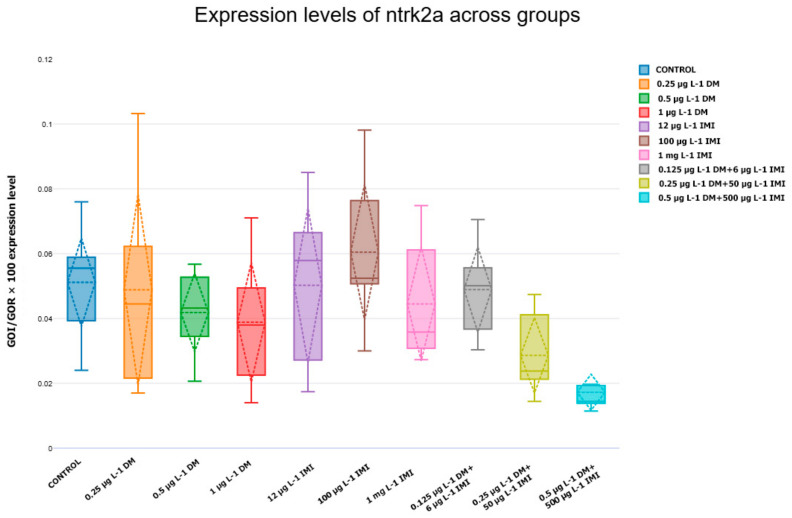
Expression levels of *ntrk2a* across experimental groups. Box plot illustrating the expression of *ntrk2a* (TrkB receptor isoform) in zebrafish following exposure to DM, IMI, and their mixtures. The *y*-axis represents normalized gene expression values (*GOI*/*GOR* × 100), while the *x*-axis corresponds to different experimental conditions. Statistical differences between groups were determined using ANOVA and Tukey post hoc tests (n = six).

**Figure 6:**
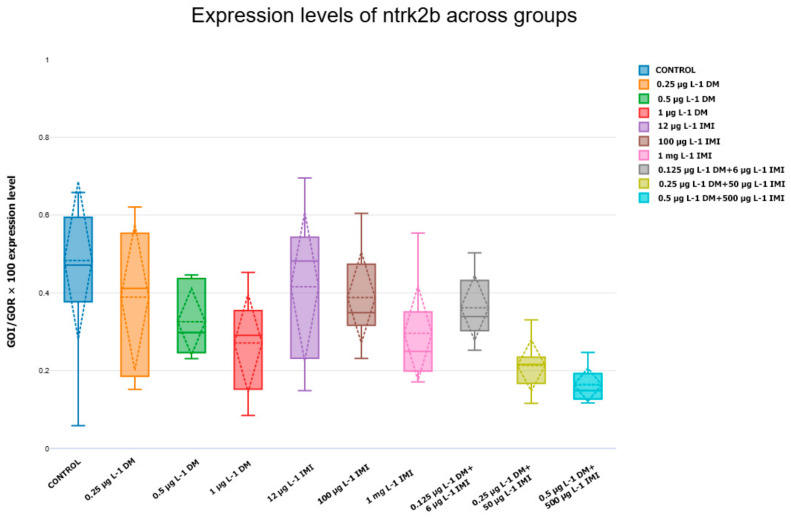
Expression levels of *ntrk2b* across experimental groups. Box plot depicting *ntrk2b* expression levels, another TrkB receptor isoform, in zebrafish under pesticide exposure. The *y*-axis shows normalized gene expression values (*GOI/GOR* × 100), and the *x*-axis denotes the experimental groups. The plot provides insights into how pesticide exposure influences TrkB receptor expression, with statistical significance assessed using ANOVA and post hoc Tukey tests (n = six).

In parallel with *BDNF* alterations, the expression of its receptors, TrkB (*ntrk2a* and *ntrk2b*), followed similar trends (Figure 5 and Figure 6). The control group displayed consistent expression levels, which were used as a baseline reference.

For ntrk2a, low-dose pesticide exposure did not lead to significant changes in expression, while moderate doses of DM and IMI induced slight fluctuations. However, at higher concentrations (1 µg L^−1^ DM and 1000 µg L^−1^ IMI), ntrk2a expression was significantly reduced (*p* < 0.01, ANOVA with Tukey post hoc test). The most pronounced downregulation occurred in the combined exposure groups, particularly in 0.5 µg L^−1^ DM + 500 µg L^−1^ IMI, which showed the lowest ntrk2a expression values (*p* < 0.001).

The expression of *ntrk2b* followed a comparable pattern, with high-dose DM and IMI exposures leading to a statistically significant decrease in expression compared to the control (*p* < 0.05). The mixed-exposure groups again exhibited the most severe suppression of ntrk2b, with 0.5 µg L^−1^ DM + 500 µg L^−1^ IMI displaying the lowest expression levels among all conditions (*p* < 0.0001, Tukey test).

#### 3.3.2. *NGF* and *TrkA* Expression

**Figure 7:**
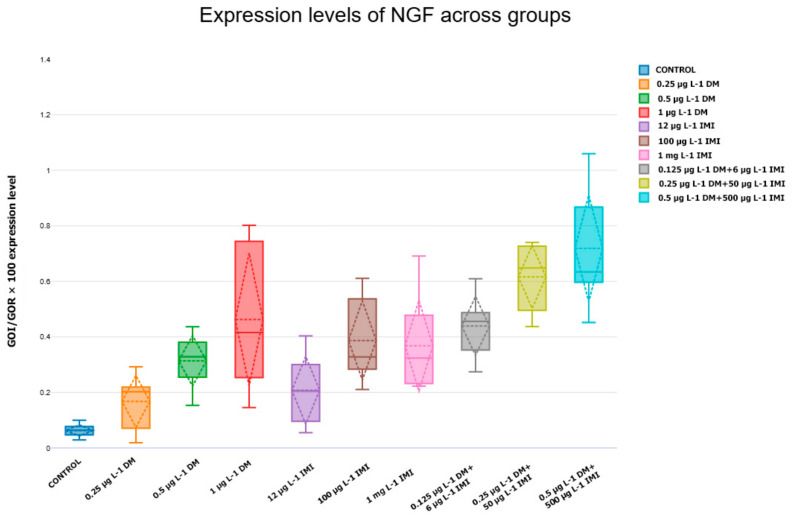
Expression levels of *NGF* across experimental groups—the relative expression levels of *NGF* in zebrafish exposed to different concentrations of DM, IMI, and their combinations (n = six per group). The *y*-axis indicates normalized gene expression (*GOI/GOR* × 100), while the *x*-axis represents the experimental groups. Each box plot displays the mean (horizontal discontinued line), median (horizontal continued line), interquartile range (box), and data variability (whiskers), with individual data points shown. Statistical analysis was performed using ANOVA followed by Tukey post hoc tests, with significance set at *p* < 0.05.

*NGF* expression varied across exposure groups, showing both dose-dependent increases and differential responses between single and combined pesticide exposures (Figure 7). The control group exhibited stable *NGF* expression, serving as a baseline for comparison.

Exposure to low concentrations of DM (0.25 µg L^−1^ and 0.5 µg L^−1^) resulted in a moderate increase in *NGF* expression, though these changes were not statistically significant compared to the control (*p* > 0.05, n = six). However, at 1 µg L^−1^ DM, *NGF* expression increased significantly, with a higher median value than all other single-exposure groups (*p* < 0.01, Tukey test). This suggests a potential compensatory response to neurotoxic stress at elevated DM concentrations.

IMI exposure produced more variable expression patterns. While 12 µg L^−1^ and 100 µg L^−1^ IMI groups showed no significant changes compared to the control (*p* > 0.05), 1000 µg L^−1^ IMI resulted in a slight increase in *NGF* expression, although statistical significance was not reached.

A different trend was observed in combined pesticide exposure groups, where *NGF* expression was significantly higher than in both control and single-exposure groups. The 0.25 µg L^−1^ DM + 50 µg L^−1^ IMI group exhibited marked upregulation of *NGF* expression (*p* < 0.05), while the 0.5 µg L^−1^ DM + 500 µg L^−1^ IMI group displayed the highest *NGF* expression levels of all conditions (*p* < 0.001). These findings suggest that combined pesticide exposure may trigger a stronger neurotrophic response, potentially as an adaptive mechanism to counteract neurotoxic damage.

**Figure 8:**
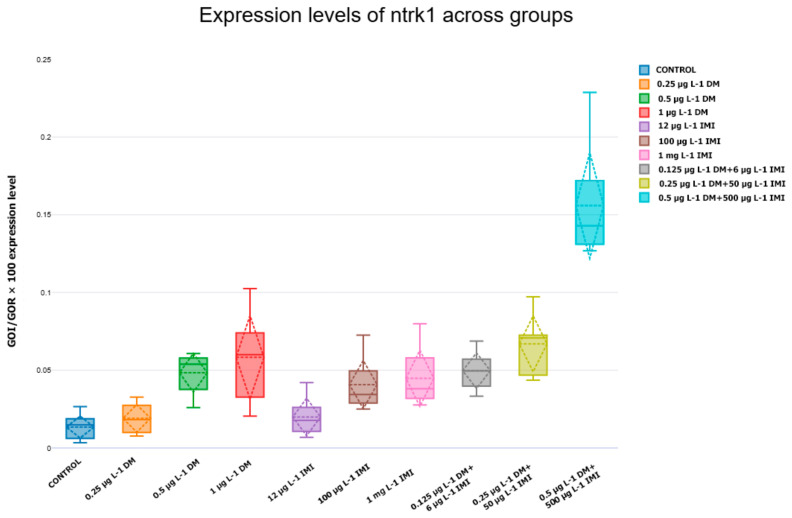
Expression levels of *ntrk1* across experimental groups. Box plot illustrating the expression of *ntrk1* (TrkA receptor) in zebrafish following exposure to different pesticide conditions (n = six per group). The *y*-axis represents normalized gene expression (*GOI/GOR* × 100), while the *x*-axis corresponds to the experimental groups. Statistical significance was determined using ANOVA and Tukey post hoc tests, with differences considered significant at *p* < 0.05. The data indicate alterations in *ntrk1* expression in response to pesticide exposure, with potential implications for neurotrophic signaling.

TrkA (*ntrk1*) expression followed a similar dose-dependent trend to NGF (Figure 8). The control group maintained stable *ntrk1* expression, providing a reference for pesticide-exposed groups.

Exposure to low DM concentrations (0.25 µg L^−1^ and 0.5 µg L^−1^) resulted in a slight, non-significant increase in *ntrk1* expression. However, at 1 µg L^−1^ DM, *ntrk1* expression was significantly upregulated compared to the control (*p* < 0.05), suggesting a potential neuroprotective response at this dose.

IMI exposure at low and moderate concentrations (12 µg L^−1^ and 100 µg L^−1^) did not significantly alter *ntrk1* expression. However, at 1000 µg L^−1^ IMI, a modest but non-significant increase was observed, potentially reflecting a compensatory mechanism in response to neuronal stress.

In the combined pesticide exposure groups, *ntrk1* expression exhibited the most pronounced upregulation. The 0.25 µg L^−1^ DM + 50 µg L^−1^ IMI group showed a statistically significant increase in expression compared to control and single-exposure groups (*p* < 0.05). The 0.5 µg L^−1^ DM + 500 µg L^−1^ IMI group displayed the highest *ntrk1* expression levels, with significant upregulation compared to all other groups (*p* < 0.001). This suggests that co-exposure to DM and IMI may enhance TrkA receptor signaling, potentially as an attempt to mitigate pesticide-induced neurotoxic stress.

#### 3.3.3. Expression Levels of *ntf-3*, *ntf-4/5*, *ntrk3a*, and *ntrk3b*

**Figure 9:**
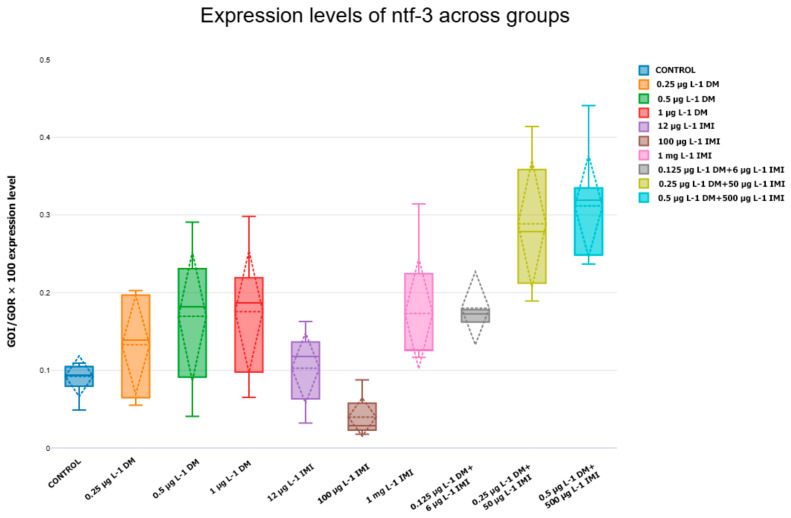
Expression levels of *ntf-3* across experimental groups. Box plot illustrating *ntf-3* expression levels (n = six per group) in zebrafish exposed to DM, IMI, and their mixtures. Statistical significance was determined using ANOVA and Tukey post hoc tests.

The analysis of *ntf-3* expression revealed a dose-dependent increase in response to DM exposure, particularly at higher concentrations (Figure 9). While low and moderate doses of DM (0.25 µg L^−1^ and 0.5 µg L^−1^) resulted in a mild, non-significant increase compared to the control, exposure to 1 µg L^−1^ DM led to a statistically significant upregulation of *ntf-3* (*p* < 0.01, Tukey post hoc test, n = six per group). This suggests that higher concentrations of DM may trigger compensatory neurotrophic signaling as a response to neurotoxic stress. In contrast, exposure to IMI at 12 µg L^−1^ and 100 µg L^−1^ did not induce significant changes in *ntf-3* expression, whereas 1000 µg L^−1^ IMI resulted in a mild downregulation, though this effect was not statistically significant. Notably, the combination of 0.25 µg L^−1^ DM and 50 µg L^−1^ IMI induced a significant increase in *ntf-3* expression (*p* < 0.05), and this effect was further pronounced in the 0.5 µg L^−1^ DM + 500 µg L^−1^ IMI group, which exhibited the highest *ntf-3* expression levels among all experimental conditions (*p* < 0.001). These findings suggest that co-exposure to DM and IMI may lead to an enhanced neurotrophic response, potentially as a mechanism to counteract neurotoxic effects.

**Figure 10:**
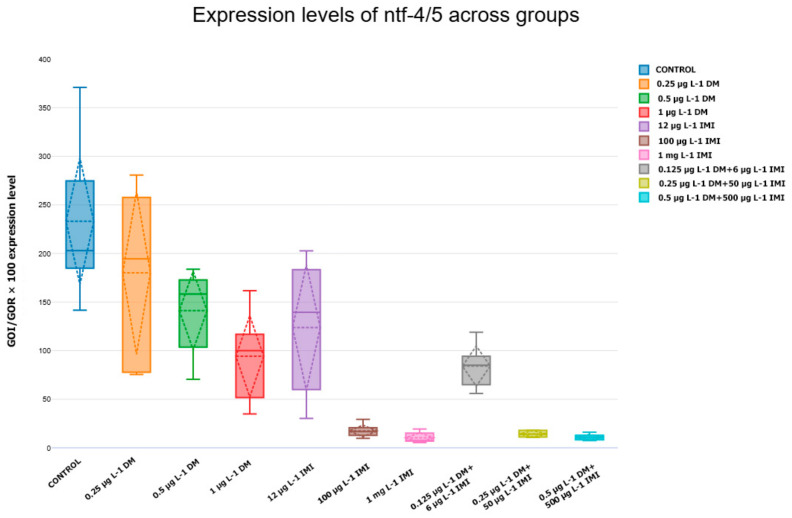
Expression levels of *ntf-4/5* across experimental groups. Box plot showing *ntf-4/5* expression changes (n = six per group) across experimental conditions. Statistical comparisons were performed using ANOVA and Tukey post hoc tests.

In contrast to *ntf-3*, the expression pattern of *ntf-4/5* followed a distinct trajectory (Figure 10). While low-dose DM exposure (0.25 µg L^−1^ and 0.5 µg L^−1^) did not significantly alter *ntf-4/5* levels, exposure to 1 µg L^−1^ DM caused a moderate, yet non-significant, reduction in expression compared to the control (*p* > 0.05). The effects of IMI exposure were more pronounced, with 100 µg L^−1^ and 1000 µg L^−1^ IMI significantly reducing *ntf-4/5* expression (*p* < 0.01, Tukey test). This downregulation was even more evident in the combined pesticide exposure groups, where 0.5 µg L^−1^ DM + 500 µg L^−1^ IMI resulted in the most significant suppression of *ntf-4/5* (*p* < 0.001). These findings indicate that while DM alone does not strongly affect *ntf-4/5* expression, IMI exposure and co-exposure to both pesticides induce a marked reduction, suggesting that these conditions may interfere with neurotrophin-mediated signaling pathways involved in neuronal maintenance.

**Figure 11:**
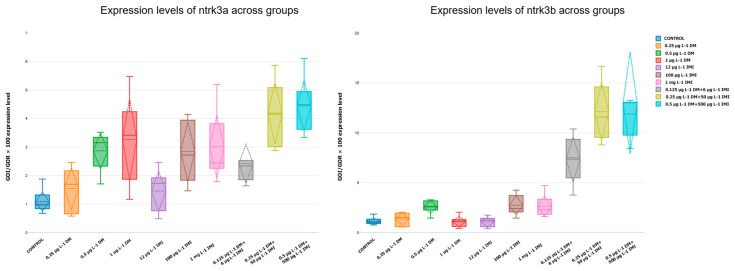
Expression levels of *ntrk3a* (**left**) and *ntrk3b* (**right**) across experimental groups. Box plots displaying *ntrk3a* and *ntrk3b* expression levels (n = six per group). The data indicate significant downregulation in IMI and mixed-exposure groups (*p* < 0.0001).

The analysis of *ntrk3a* and *ntrk3b*, the receptors for ntf-3, provided further insight into the effects of pesticide exposure on neurotrophic signaling. Expression of *ntrk3a* followed a similar pattern to *ntf-3*, with low and moderate DM exposure (0.25 µg L^−1^ and 0.5 µg L^−1^) leading to a slight but non-significant increase in expression (Figure 11). However, at 1 µg L^−1^ DM, *ntrk3a* and *ntrk3b* were significantly upregulated compared to the control (*p* < 0.05, Tukey test), suggesting that this concentration may induce a compensatory response at the receptor level. In contrast, exposure to IMI at 12 µg L^−1^ and 100 µg L^−1^ did not result in significant changes, while 1000 µg L^−1^ IMI caused a slight, though non-significant, downregulation. The effects of combined pesticide exposure were particularly notable, as the 0.5 µg L^−1^ DM + 500 µg L^−1^ IMI group exhibited the highest *ntrk3a* expression levels, significantly exceeding all other groups (*p* < 0.001) (Figure 11). These findings imply that co-exposure to DM and IMI may amplify TrkC receptor signaling, potentially in response to increased neurotoxic stress.

### 3.4. Histopathological Changes

#### 3.4.1. Histological Impairments of the Zebrafish Brain After DM Exposure

To investigate the neurotoxic effects of DM, IMI, and their combinations, histological analysis was performed on zebrafish brain sections. Luxol Fast Blue-Cresyl Violet (LFB-Cr. V) staining provided a detailed visualization of neuronal integrity, allowing for the differentiation between gray and white matter and the identification of cellular damage (Supplementary Data).

The results revealed a progressive pattern of neurodegeneration, with the severity of alterations increasing in a dose-dependent manner, particularly in groups exposed to DM and IMI combinations.

**Figure 12:**
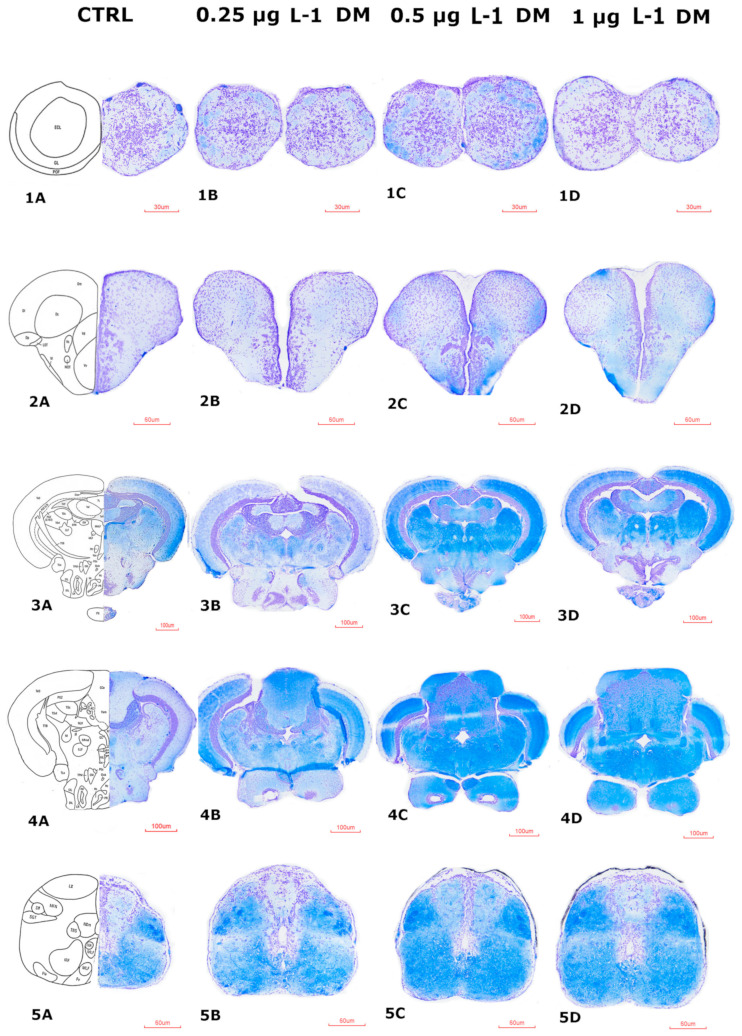
Histological sections of key brain regions of control and DM-exposed fish. Representative cross-sections illustrating the structural organization of different brain regions in zebrafish from the control group and those exposed to increasing concentrations of deltamethrin (DM). Panels (**1A**–**5A**) correspond to the control group, while (**1B**–**5B**,**1C**–**5C**,**1D**–**5D**) depict sections from fish exposed to 0.25 µg L^−1^, 0.5 µg L^−1^, and 1 µg L^−1^ DM, respectively. The images display the following brain regions: Olfactory bulbs (**1A**–**1D**), scale bar = 30 µm; Telencephalon (**2A**–**2D**), scale bar = 60 µm; Diencephalon and mesencephalon (**3A**–**3D**), scale bar = 100 µm; Rhombencephalon (**4A**–**4D**), scale bar = 100 µm; Medulla oblongata (**5A**–**5D**), scale bar = 60 µm. These histological sections highlight potential structural alterations associated with different pesticide exposures. Changes in tissue integrity, cellular arrangement, and neurotoxic effects are examined across experimental conditions.

Brain sections from the control group (Figure 12, 1A**→**5A) exhibited normal histological architecture, with clearly defined neuronal layers, intact ventricles, and uniform cellular distribution across all regions. No evidence of cellular disorganization, degeneration, or vacuolation was observed.

In contrast, DM-exposed groups displayed a spectrum of neurotoxic effects, ranging from mild structural disorganization to extensive neuronal degeneration (Figure 12).

At 0.25 µg L^−1^ DM exposure, mild vacuolization, and slight neuronal shrinkage were observed (Figure 12, 1B**→**5B). The organization of the brain regions remained largely preserved, though a slight increase in intercellular spaces was detected. The midbrain and hindbrain regions (Figure 12, 2B, 3B) showed minimal disorganization compared to the control.

Exposure to 0.5 µg L^−1^ DM induced moderate histopathological change (Figure 12, 1C**→**5C). Increased vacuolization, neuronal pyknosis (condensed nuclei), increased cytoplasmic eosinophilia, and occasionally disrupted layering in the cerebellum and forebrain were evident (Figure 12, 3C, 4C). The periventricular zones displayed cellular degeneration and reduced density of neuronal populations as early signs of neuroinflammation.

At the highest exposure (1 µg L^−1^), more significant histopathological alterations were detected across brain regions (Figure 12, 1D**→**5D). Marked neuronal loss, substantial vacuolization, and widespread gliosis were evident (Figure 12, 4D, 5D). Cerebellar architecture appeared disrupted, with neuronal necrosis and cellular debris observed in the hindbrain (Figure 12, 5D).

#### 3.4.2. Histological Impairments of the Zebrafish Brain After IMI Exposure

No relevant modifications of the histological structure of the brain regions were observed in all of the IMI-exposed zebrafish (12 µg/L^−1^ IMI, 100 µg/L^−1^ IMI, and 1000 µg L^−1^ IMI).

#### 3.4.3. Histological Impairments of the Zebrafish Brain After DM-IMI Exposure

**Figure 13:**
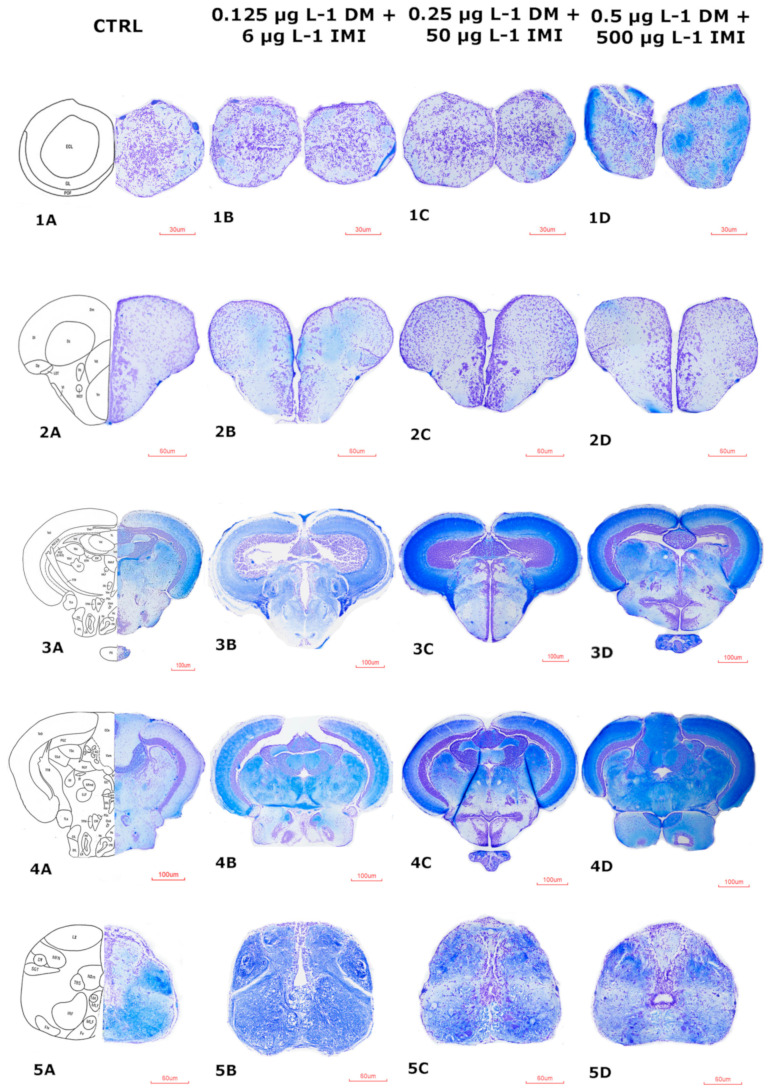
Representative histological sections illustrating structural organization in various brain regions of zebrafish from the control group and those exposed to different concentrations of deltamethrin (DM) and imidacloprid (IMI) in combination. Panels (**1A**–**5A**) correspond to the control group, while (**1B**–**5B**,**1C**–**5C**,**1D**–**5D**) show sections from zebrafish exposed to 0.125 µg L^−1^ DM + 6 µg L^−1^ IMI, 0.25 µg L^−1^ DM + 50 µg L^−1^ IMI, and 0.5 µg L^−1^ DM + 500 µg L^−1^ IMI, respectively. The images display the following brain regions: Olfactory bulbs (**1A**–**1D**), scale bar = 30 µm; Telencephalon (**2A**–**2D**), scale bar = 60 µm; Diencephalon and mesencephalon (**3A**–**3D**), scale bar = 100 µm; Rhombencephalon (**4A**–**4D**), scale bar = 100 µm; Medulla oblongata (**5A**–**5D**), scale bar = 60 µm.

Co-exposure to DM and IMI resulted in more severe histopathological changes compared to DM alone, suggesting a synergistic toxic effect (Figure 13). Minimal histopathological changes were observed at the lowest concentration of the mixture (0.125 µg L^−1^ DM + 6 µg L^−1^ IMI). Slight neuronal shrinkage and mild periventricular vacuolization were noted, primarily in the midbrain and hindbrain (Figure 13, 3B, 4B). Cellular architecture remained largely intact, suggesting early neuroinflammation or oxidative stress signs.

Moderate neurodegenerative changes became apparent in the group exposed to 0.25 µg L^−1^ DM + 50 µg L^−1^ IMI. Increased neuronal pyknosis, vacuolization, and gliosis were evident across the midbrain and hindbrain regions (Figure 13, 3C, 4C). The cerebellum displayed disrupted layering, and cellular density appeared reduced.

At 0.125 µg L^−1^ DM + 6 µg L^−1^ IMI, the histopathological features were comparable to those of the 0.25 µg L^−1^ DM group, with mild vacuolization and scattered neuronal condensation. However, at 0.25 µg L^−1^ DM + 50 µg L^−1^ IMI, the diencephalon and mesencephalon exhibited extensive perineuronal edema and significant vacuolization, suggesting an exacerbated cytotoxic effect due to pesticide interaction. Important histopathological alterations were observed at the highest co-exposure level (0.5 µg L^−1^ DM + 500 µg L^−1^ IMI). Marked neuronal necrosis, extensive vacuolization, and widespread gliosis were detected throughout all brain regions (Figure 13, 4D, 5D). The cerebellum exhibited significant atrophy, and large periventricular spaces indicated substantial neuronal loss. The forebrain also presented evidence of cellular debris and severe architectural disorganization. The presence of pale-stained, swollen neurons with highly condensed nuclei further supports the hypothesis of synergistic neurotoxicity, potentially leading to functional impairments in motor control and sensory processing.

## 4. Discussions

The present study systematically investigated the neurotoxic effects of deltamethrin (DM), imidacloprid (IMI) and their combinations in zebrafish, focusing on behavioral, molecular, and histological alterations. The results provide compelling evidence that both pesticides, individually and in combination, induce significant changes in neurobehavioral responses, gene expression patterns, and brain histology, with the co-exposure groups exhibiting the most pronounced effects. The findings contribute to a deeper understanding of pesticide-induced neurotoxicity and emphasize the potential risks associated with simultaneous exposure to DM and IMI, which are commonly detected in aquatic environments.

Behavioral assessments revealed a dose-dependent increase in neurotoxic symptoms following exposure to DM and IMI, with the highest severity observed in the combined exposure groups. Zebrafish exposed to higher DM concentrations (1 µg L^−1^) and combined DM-IMI treatments exhibited significant behavioral alterations, including hyperactivity bursts, erratic movements, thigmotaxis, reduced exploration, and motor incoordination. These findings align with previous studies demonstrating that pyrethroids such as DM exert strong neurotoxic effects by disrupting voltage-gated sodium channels, leading to neuronal hyperexcitability [93]. The observed hyperactivity at lower DM concentrations may reflect an initial compensatory response to increased synaptic activity. In contrast, higher concentrations lead to neuromuscular dysfunction and reduced mobility, indicative of a shift from excitotoxicity to neuronal suppression.

IMI exposure alone resulted in more variable behavioral outcomes, with higher doses (1000 µg L^−1^) inducing motor impairments, immobility, and reduced feeding. As a neonicotinoid insecticide, IMI primarily targets nicotinic acetylcholine receptors (nAChRs), leading to excessive synaptic excitation and potential receptor desensitization [73]. These mechanisms may explain the progressive decline in locomotion and feeding behaviors observed in the higher IMI exposure groups.

Co-exposure to DM and IMI elicited the most severe behavioral impairments, suggesting potential synergistic interactions between the two pesticides. The 0.5 µg L^−1^ DM + 500 µg L^−1^ IMI group exhibited the highest behavioral severity scores, with significant neuromuscular dysfunction. The combined effects may stem from the concurrent disruption of sodium channel activity (DM) and cholinergic neurotransmission (IMI), leading to compounded neurotoxicity that exceeds the effects of individual pesticide exposure.

The observed mortality trends reinforce the dose-dependent neurotoxicity of DM and IMI. Mortality rates increased progressively over time, with the highest lethality observed in the combined pesticide exposure groups. The 0.5 µg L^−1^ DM + 500 µg L^−1^ IMI group reached a mortality rate of 30% by the end of the 21-day exposure period, significantly exceeding the rates observed in single-exposure groups. This pattern is consistent with findings that DM and IMI exert toxic effects through different but complementary mechanisms, exacerbating oxidative stress, synaptic dysfunction, and neuroinflammation when combined. The linear regression analysis confirmed a significant association between increasing pesticide concentration and cumulative mortality, highlighting the potential risks of prolonged environmental exposure.

The analysis of brain-derived neurotrophic factor (*BDNF*) and its receptor TrkB (*ntrk2a, ntrk2b*) revealed significant downregulation in response to pesticide exposure, particularly in the co-exposure groups. *BDNF* plays a crucial role in synaptic plasticity, neuronal survival, and cognitive function, and its disruption is commonly associated with neurodegenerative diseases and pesticide-induced neurotoxicity. The significant reduction of *BDNF* expression in the 0.5 µg L^−1^ DM + 500 µg L^−1^ IMI group (*p* < 0.0001) suggests that co-exposure to DM and IMI interferes with neurotrophic support mechanisms, potentially leading to neuronal damage.

A similar pattern was observed in the expression of TrkB receptors (*ntrk2a and ntrk2b*), with high-dose pesticide exposure leading to significant downregulation. Since *BDNF*-TrkB signaling is essential for neurogenesis and synaptic maintenance, these findings suggest that chronic exposure to DM and IMI may impair brain plasticity and neurodevelopment, increasing susceptibility to cognitive deficits and neurodegeneration.

The results of this study demonstrate that both *BDNF* and its receptors (TrkB, encoded by *ntrk2a* and *ntrk2b*) are significantly affected by exposure to DM, IMI, and their combinations. While low pesticide doses appeared to induce mild compensatory effects, higher concentrations led to progressive downregulation of neurotrophic signaling, suggesting a shift from adaptation to neurotoxicity.

IMI exposure alone produced variable effects, but at its highest concentration (1000 µg L^−1^), it significantly reduced *BDNF* and TrkB expression, confirming its neurotoxic potential. The most severe reductions in gene expression were observed in the combined pesticide exposure groups, particularly in 0.5 µg L^−1^ DM + 500 µg L^−1^ IMI, where *BDNF*, *ntrk2a*, and *ntrk2b* were all significantly downregulated compared to controls and single-exposure groups (*p* < 0.0001).

In contrast to *BDNF*, nerve growth factor (*NGF*) and its receptor TrkA (*ntrk1*) exhibited an upregulation in response to pesticide exposure, particularly in the combined exposure groups. The significant increase in NGF expression in the 0.5 µg L^−1^ DM + 500 µg L^−1^ IMI group (*p* < 0.001) suggests a compensatory neuroprotective response to counteract pesticide-induced neuronal damage. However, sustained upregulation of *NGF* can lead to dysregulated neurotrophic signaling, potentially contributing to aberrant synaptic remodeling and neuroinflammation.

The results indicate that *NGF* and *ntrk1* expression levels are significantly influenced by exposure to DM, IMI, and their combinations, with higher concentrations leading to increased neurotrophic factor and receptor expression. While low-dose pesticide exposure did not significantly alter expression, higher concentrations—particularly 1 µg L^−1^ DM—induced significant upregulation, likely in response to neurotoxic effects.

IMI exposure alone produced variable effects, but at its highest concentration (1000 µg L^−1^), a slight increase in *NGF* and *ntrk1* expression was observed, though statistical significance was not reached.

A similar compensatory response was observed in *ntf-3* and its receptor TrkC (*ntrk3a* and *ntrk3b*), both of which were significantly upregulated in the co-exposure groups. In contrast, *ntf-4/5*, *ntrk2a*, and *ntrk2b* were significantly downregulated, particularly in high-dose IMI and combined pesticide exposure groups. These findings suggest that IMI may selectively impair certain neurotrophic pathways while triggering compensatory mechanisms in others, leading to an imbalance in neurotrophic regulation.

The results indicate that exposure to DM and IMI influences neurotrophic signaling in a gene-specific and dose-dependent manner. While DM exposure alone led to an upregulation of *ntf-3* and *ntrk3a/b* at higher concentrations, *ntf-4/5* and *ntrk2a/b* were largely unaffected. In contrast, IMI exposure resulted in significant downregulation of *ntf-4/5* and *ntrk2a/b* at higher doses, suggesting that IMI may have a more potent suppressive effect on neurotrophic receptor signaling. The most significant alterations were observed in combined pesticide exposure groups, where *ntf-3* and *ntrk3a/b* were significantly upregulated, whereas *ntf-4/5* and *ntrk2a/b* were significantly downregulated. These findings suggest that co-exposure to DM and IMI exerts distinct regulatory effects on neurotrophin signaling pathways, with potential implications for neuronal function and development. Further investigation is required to determine the long-term consequences of these regulatory changes on neurodevelopmental processes.

The observed downregulation of *BDNF* in zebrafish brains following exposure to DM and IMI, individually and in combination, aligns with existing literature indicating the neurotoxic effects of pesticides on neurotrophin signaling pathways. *BDNF* is a critical mediator of neuronal survival, differentiation, and synaptic plasticity, and its reduced expression has been associated with neurodegeneration and cognitive deficits in various models [94,95].

The dose-dependent reduction in *BDNF* expression in DM-treated groups corroborates previous findings demonstrating that pyrethroid insecticides induce oxidative stress and apoptosis in neural tissues [96]. Deltamethrin, in particular, disrupts calcium homeostasis and activates apoptotic pathways, possibly contributing to *BDNF* transcription suppression [97] IMI, a neonicotinoid insecticide, has similarly been implicated in neurotoxicity through its interaction with nicotinic acetylcholine receptors (nAChRs) [98]. The most striking observation was the pronounced reduction in *BDNF* expression in groups exposed to mixtures of DM and IMI. This synergistic effect likely reflects the convergence of distinct neurotoxic mechanisms, amplifying cellular stress and impairing neurotrophic signaling more severely than individual exposures.

The strong correlation between the downregulation of *BDNF* and the reduced expression of *ntrk2a* and *ntrk2b* supports the hypothesis that pesticide exposure disrupts the BDNF-TrkB signaling pathway in zebrafish brains. This signaling cascade plays a pivotal role in neurogenesis, synaptic plasticity, and neuronal survival, and its impairment may contribute to the neurotoxic effects induced by pesticides [99,100].

The dose-dependent reduction in *ntrk2* expression suggests that pesticide exposure directly affects receptor availability, diminishing neurons’ capacity to respond to *BDNF*. This aligns with previous findings that demonstrate TrkB receptors’ vulnerability to oxidative stress and apoptotic signals triggered by environmental contaminants [101,102,103,104]. The observed results underscore the importance of evaluating neurotoxic effects at the level of neurotrophins and through the analysis of receptor expression. The simultaneous downregulation of *BDNF* and TrkB receptors highlights the potential for severe impairments in neurotrophic signaling, raising concerns about long-term cognitive and behavioral deficits in zebrafish populations exposed to environmental contaminants. The significant upregulation of *NGF* and its receptor ntrk1 in zebrafish brains following pesticide exposure highlights the activation of neuroprotective and regenerative pathways. *NGF* plays a crucial role in neuronal survival, differentiation, and synaptic plasticity, and its increased expression likely reflects a compensatory mechanism aimed at mitigating pesticide-induced neurotoxicity [105,106,107].

The histological analysis further supports the neurotoxic effects of DM and IMI, particularly in the combined exposure groups. Significant structural alterations were observed in the olfactory bulbs, telencephalon, diencephalon, mesencephalon, rhombencephalon, and medulla oblongata. The most notable changes included increased vacuolization, neuronal degeneration, and tissue disorganization, particularly in the 1 µg L^−1^ DM and 0.5 µg L^−1^ DM + 500 µg L^−1^ IMI groups. These observations align with previous studies showing that pyrethroids and neonicotinoids can induce oxidative stress and apoptotic pathways in neural tissues.

The most severe histopathological changes were observed in the co-exposure groups, reinforcing the hypothesis that simultaneous exposure to DM and IMI exacerbates neurotoxic damage. The increased vacuolization and neuronal loss observed in high-dose groups suggest that long-term exposure could lead to irreversible neurodegeneration.

The findings of this study have significant implications for environmental safety and public health. The widespread use of DM and IMI in agricultural and urban settings has led to their frequent detection in aquatic ecosystems, raising concerns about potential neurotoxic effects on non-target species. The evidence presented here indicates that chronic exposure to these pesticides, even at environmentally relevant concentrations, can induce significant neurobehavioral and molecular alterations.

The severity of histopathological alterations corresponded with behavioral and molecular findings, providing multilevel evidence of neurotoxicity. Regions exhibiting the most pronounced structural damage, such as the medulla oblongata and rhombencephalon, align with observed deficits in locomotion and increased behavioral severity scores. Moreover, the increased expression of neuroinflammatory markers and dysregulated neurotrophin signaling further supports the hypothesis that DM and IMI induce neurodegeneration via oxidative stress and excitotoxicity.

The histopathological alterations observed in zebrafish brains exposed to increasing concentrations of DM correlate with molecular changes in neurotrophin gene expression, as shown in previous experiments. Elevated expression of NGF and ntf-3 at lower DM concentrations (0.25–0.5 µg L^−1^) suggests an initial compensatory neuroprotective response to oxidative stress and neuronal damage [108]. This aligns with reports that neurotrophins are upregulated during early neurotoxic stress to promote neuronal survival and repair [108,109,110]. However, at 1 µg L^−1^ DM, significant downregulation of neurotrophin expression (especially ntf-4/5) coincided with extensive neuronal degeneration and histopathological damage. This decline indicates prolonged or high-dose pesticide exposure surpasses the neuroprotective threshold, leading to apoptosis, synaptic loss, and impaired neurogenesis.

The role of ntrk receptors in mediating neurotrophin signaling also supports these findings. Diminished expression of *ntrk2a/b* and *ntrk3a* receptors at higher DM concentrations may exacerbate neuronal vulnerability by limiting neurotrophin signaling pathways essential for synaptic plasticity and neuronal differentiation [111,112].

Behavioral deficits (increased hyperactivity, erratic movements, reduced exploration) were most severe in groups with the most pronounced downregulation of *BDNF* and *NGF*, suggesting that neurotrophin depletion contributes to motor and cognitive dysfunctions [113].

Histopathological alterations (neuronal shrinkage, vacuolization, gliosis) were most evident in regions where neurotrophin expression was dysregulated, particularly in the telencephalon, mesencephalon, and rhombencephalon, indicating a direct link between molecular and structural damage [114].

These findings align with previous studies in rodent models of pesticide exposure, where *BDNF* depletion and neuroinflammation were linked to hippocampal atrophy and memory deficits [115]. These studies further support the translational relevance of our zebrafish model [116].

Several mechanisms may contribute to pesticide-induced neurotrophin alterations: oxidative stress and neuroinflammation, excitotoxicity and calcium dysregulation, or failure of neuroprotective compensation.

Pesticides like DM and IMI have been shown to increase reactive oxygen species (ROS) production, leading to oxidative DNA damage and lipid peroxidation, which can disrupt *BDNF* and *NGF* transcriptional regulation [117]. Elevated ROS levels may also activate pro-inflammatory pathways, further exacerbating neurotrophin depletion [118]. Strungaru et al. (2019) conducted a study to assess the chronic effects of DM exposure on zebrafish (*Danio rerio*), focusing on behavioral changes, neurotoxicity, and alterations in elemental concentrations within the fish body [119]. Histological examinations revealed significant damage to the nervous system, specifically in the telencephalon, optic tectum, and cerebellum, across all studied DM concentrations. Immunocytochemical analyses indicated increased activity of toxicity markers such as PCNA, p53, and TUNEL, suggesting enhanced cell proliferation, apoptosis, and DNA fragmentation, respectively.

Pesticide exposure has been linked to glutamate excitotoxicity, which leads to neuronal calcium overload and downstream apoptotic signaling, further impairing BDNF-TrkB and NGF-TrkA signaling [120].

The initial upregulation of TrkC receptors (*ntrk3a/b*) and *ntf-3, ntf-4/5* respectively in some exposure groups may reflect an attempt to counteract neuronal damage. However, as pesticide toxicity progresses, these compensatory mechanisms fail, leading to irreversible neurodegeneration [34,38].

Our findings highlight pesticide exposure’s potential long-term neurotoxic effects, raising concerns for aquatic ecosystems and vertebrate neurobiology.

The disruption of neurotrophic signaling pathways in zebrafish suggests that chronic pesticide exposure may lead to long-term cognitive and behavioral deficits, which could have serious ecological consequences [72,121].

Given the parallels between zebrafish and mammalian neurodevelopment, our findings also raise concerns about pesticide-induced neurotoxicity in humans, particularly in populations chronically exposed to environmental pesticide residues [122,123,124,125,126].

These results reinforce the need for stricter environmental regulations on pesticide usage, as even low-dose exposure appears to induce significant neurotrophic and behavioral alterations.

## 5. Conclusions

This study provides comprehensive evidence of the neurotoxic effects of deltamethrin DM and IMI exposure in zebrafish, with co-exposure amplifying behavioral, molecular, and histopathological impairments. Behavioral analysis revealed hyperactivity, erratic swimming, and reduced exploratory behavior, correlating with neurotrophic gene dysregulation and structural neurodegeneration. *BDNF* and *NGF* downregulation and TrkA and TrkB suppression suggest impaired neurotrophic signaling, contributing to neuronal shrinkage, vacuolization, and gliosis observed in histological sections. These findings indicate that pesticide exposure disrupts neuronal function at multiple levels, with DM-IMI mixtures exacerbating neurotoxicity beyond individual pesticide effects.

While zebrafish provide a relevant model for neurotoxicity studies, direct extrapolation to other species requires further investigation. Additionally, although pesticide stability was controlled through regular water renewals, future studies should incorporate direct chemical quantification to confirm exposure consistency. Further research should explore oxidative stress, neuroinflammatory pathways, and apoptotic mechanisms to provide deeper insights into pesticide-induced neurotoxicity. Given the frequent detection of DM and IMI in aquatic environments, these results highlight the urgent need for regulatory reassessment of pesticide co-exposure risks and long-term neurodevelopmental effects in aquatic and terrestrial species.

## Figures and Tables

**Table 1:** Environmental conditions for housing, control, and experimental tanks.

Type of Tank	Temperature (°C)	pH	Salinity	Conductivity (μS/cm^−1^)	Ammonia (mg L^−1^)
Housing tank	25 ± 0.5	7.5	0.27	556	0.04
Control aquariums	26 ± 0.5	7.4	0.26	554	0.02
Experimental aquariums	26 ± 0.5	7.4	0.25	553	0.03

**Table 2:** The primers used for the real-time PCR (gene expression).

Gene Name	NCBI Reference Sequence	Forward Primer	Reverse Primer	Length (bp)
*ELFA*	BC064291.1	CTTCTCAGGCTGACTGTGC	CCGCTAGCATTACCCTCC	358
*BDNF*	NM_001308649.1	CTTGAGGTGGAAGGGGAAGCG	GTAACGGCGGCTCCAAAGGC	157
*NGF*	NM_199210.2	AGATGCCACGCTGGTCGATAC	ACCACCGCATGGGCTCAAC	121
*ntf-3*	NM_001327813.1	TTACCTTCATGTCGGCTCTGCTG	CGCGAGGAACATCACGTACAG	115
*ntf-4/5*	XM_005163972.4	CACGGAGGTGACAAAGAGGC	GGCGGGCTCTAGGAACGTG	79
*ntf-6/7*	NM_131064.3	CATTGAGAAGGCGGCGAAAT	CAACGACCTCATGGGCACTA	208
*ntrk1*	NM_001301356.1	GCTGCTCCTTCGCTATGCTA	TGGCGAGGTCTACATCTGTG	147
*ntrk2a*	NM_001366554.1	GTACATGATGCACGGCG	GAGATTTTCTCCGACTAGGC	217
*ntrk2b*	NM_001197161.2	CCAGAGATGTGTACAGCACC	CATTGTTTGAGAGCTGATACC	189
*ntrk3a*	NM_001256664	TCCCGAAATCAGCGTCAGTC	GGAGTGGACATTCGGTGGAT	169
*ntrk3b*	XM_002662753.6			
*ngfra*	XM_003198085	TACAGGACAGTAGGCTACAGGA	TCACACTCCTCTTCCACCGC	292
*ngfrb*	NM_001198660.1	TTAAACGGTGGAACAGTTGTAA	CTCCACTACAGTGGTCAAG	

## Data Availability

The data presented in this study are available in Supplementary Data.

## Supplementary Materials

The following supporting information can be downloaded at: https://www.mdpi.com/article/10.3390/life15040538/s1.
