# Supplementary material for: Combined Neurotoxic Effects of Commercial Formulations of Pyrethroid (Deltamethrin) and Neonicotinoid (Imidacloprid) Pesticides on Adult Zebrafish (Danio rerio): Behavioral, Molecular, and Histopathological Analysis
Source: Life (Basel). 2025 Mar 25;15(4):538. doi: 10.3390/life15040538 (PMC12028659; doi:10.3390/life15040538)
Supplement: Supplementary file 1 [file life-15-00538-s001.zip › life-3426637-supplementary.pdf]

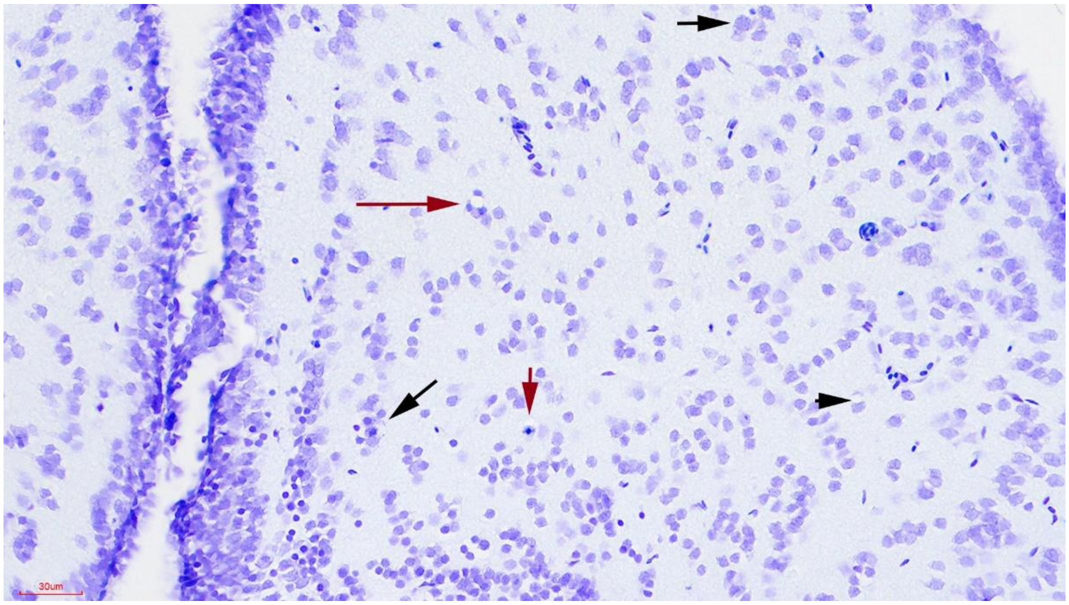

Supplementary Figure 1. Representative histological section of the zebrafish telencephalon from the DM-treated group, showing moderate neuronal damage. Mild vacuolization in the neuropil (red arrows) is indicative of potential early degenerative changes. Neurons appear slightly swollen (black arrows), but gliosis is not yet prominent. Staining: Luxol Fast Blue-Cresyl Violet. Magnification: 40x.

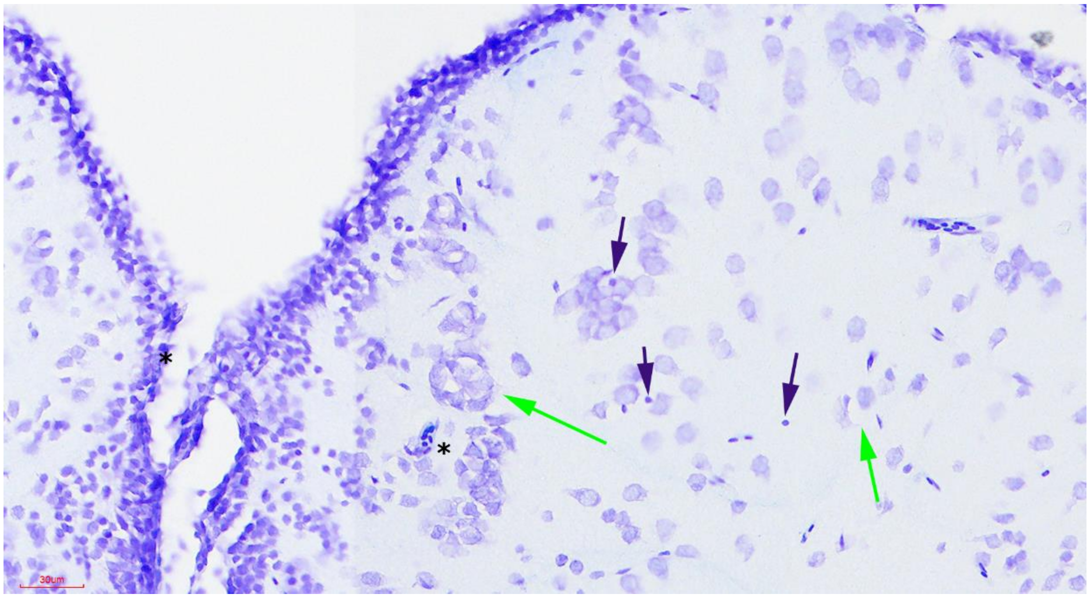

Supplementary Figure 2. Zebrafish telencephalon section from the pesticide-DM group, demonstrating initial signs of neuronal stress. Vacuolization and swollen neurons are observed (green arrows), along with scattered glial cell proliferation (violet arrows). The cytoarchitecture appears disrupted (asterisks) compared to normal conditions. Staining: Luxol Fast Blue-Cresyl Violet. Magnification: 40x.

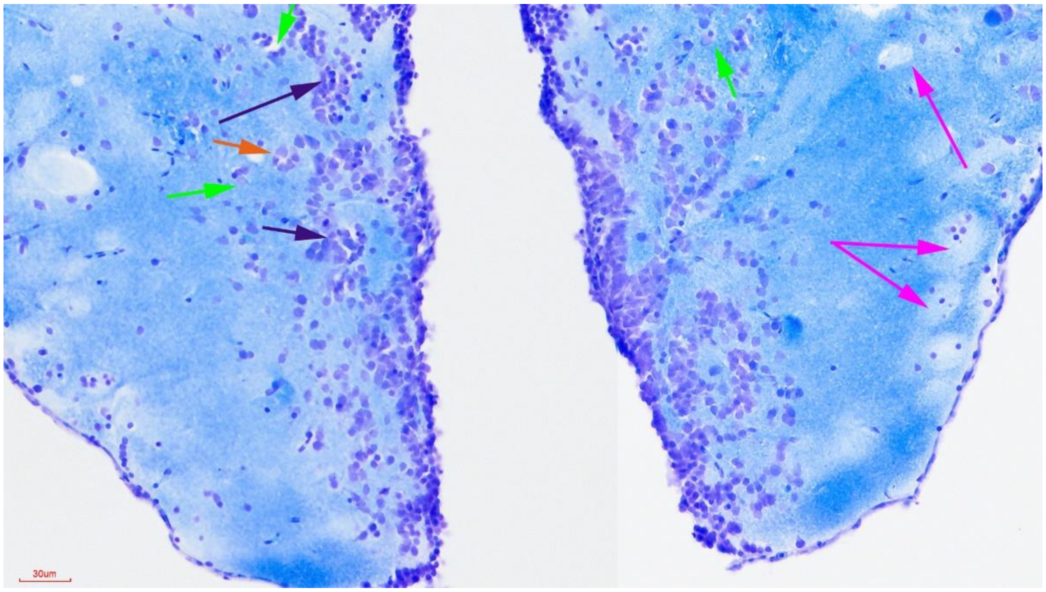

Supplementary Figure 3. Histological section of the telencephalon from the DM-IMI-treated group, showing vacuolization (green arrows), neuronal swelling (orange arrow), gliosis (violet arrows) and demyelination (pink arrows). The cytoplasmic volume of neurons is increased, and some nuclei appear pyknotic, indicating cellular stress. The surrounding neuropil shows disruption in normal structure, suggestive of gliosis. Staining: Luxol Fast Blue-Cresyl Violet. Magnification: 20x.

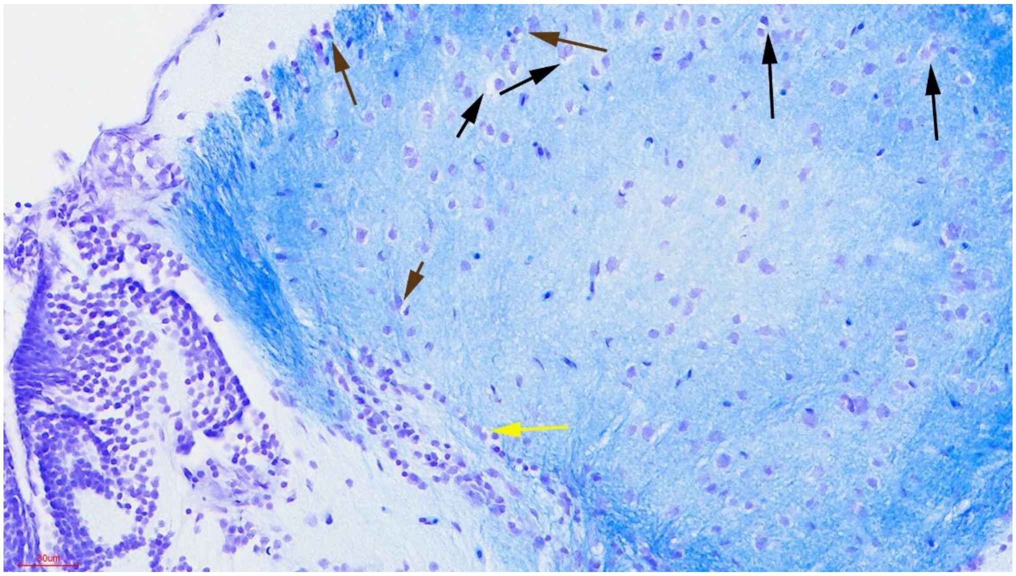

Supplementary Figure 4. Section highlighting neuronal damage in the optic tectum (diencephalon) from the DM-treated group. Shrunken neurons with vacuolization (black arrows), eosinophilic cytoplasm (yellow arrow), and nuclear condensation (brown arrows) are observed. The disruption of normal tissue architecture is apparent, with scattered reactive glial cells. Staining: Luxol Fast Blue-Cresyl Violet. Magnification: 40x.

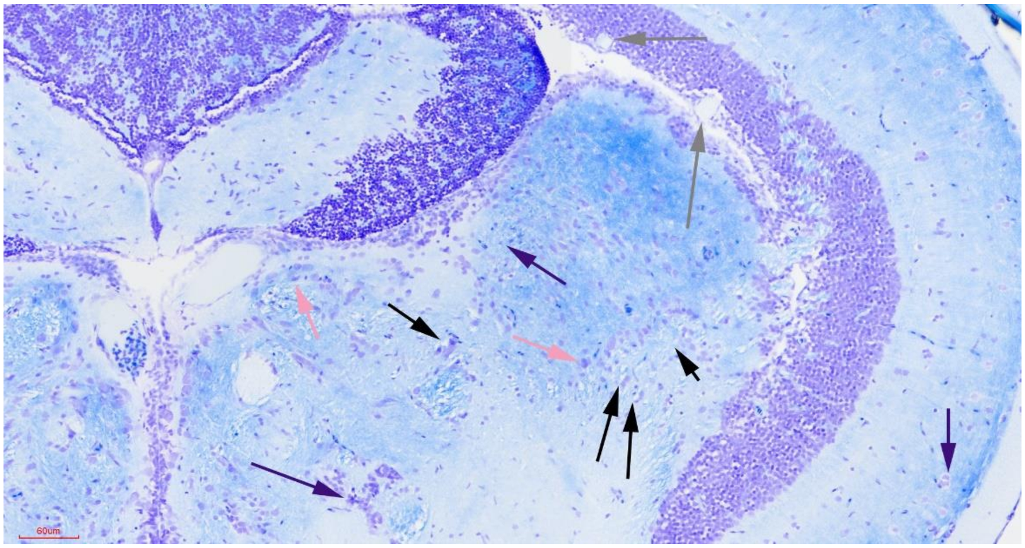

Supplementary Figure 5. Severe histopathological alterations in the optic tectum (diencephalon) from the DM-IMI-treated group, characterized by extensive vacuolation (black arrows), significant gliosis (violet arrows), and a loss of neuronal integrity (pale pink arrows). Large empty spaces (grey arrows) in the neuropil suggest severe edema or necrotic changes. The presence of reactive astrocytes indicates an inflammatory response. Staining: Luxol Fast Blue-Cresyl Violet. Magnification: 20x.

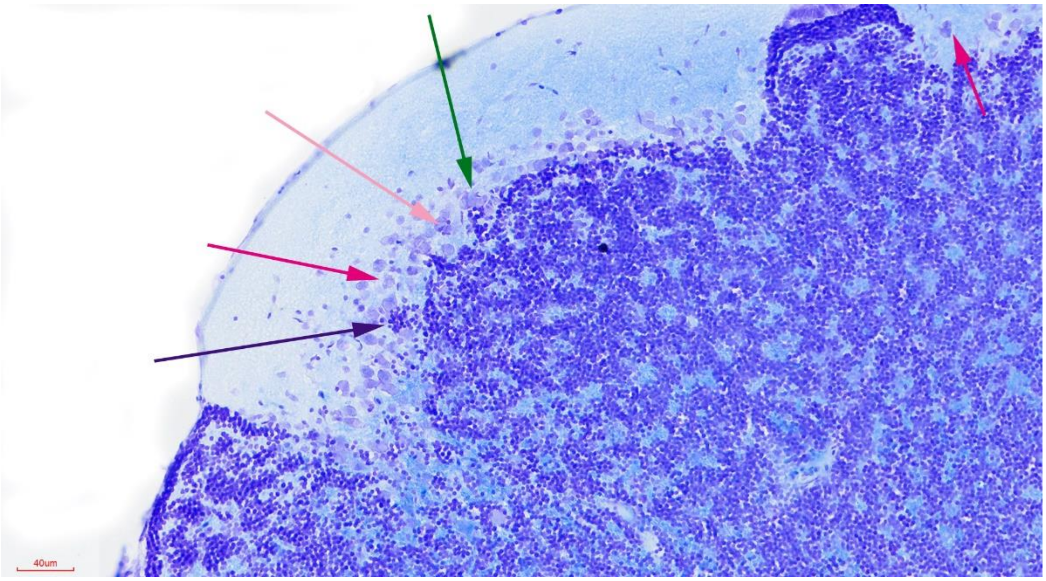

Supplementary Figure 6. Another example of extensive gliosis (violet arrow) and neurodegeneration (pale pink arrow) in the cerebellum (rhombencephalon) from the DM-IMI-treated group. Hypercellularity is observed due to an increase in reactive glial cells. The neuronal layer appears disorganized, with loss of normal cytoarchitecture (dark green arrow). Some neurons show evidence of apoptosis or necrosis (magenta arrow). Staining: Luxol Fast Blue-Cresyl Violet. Magnification: 40x

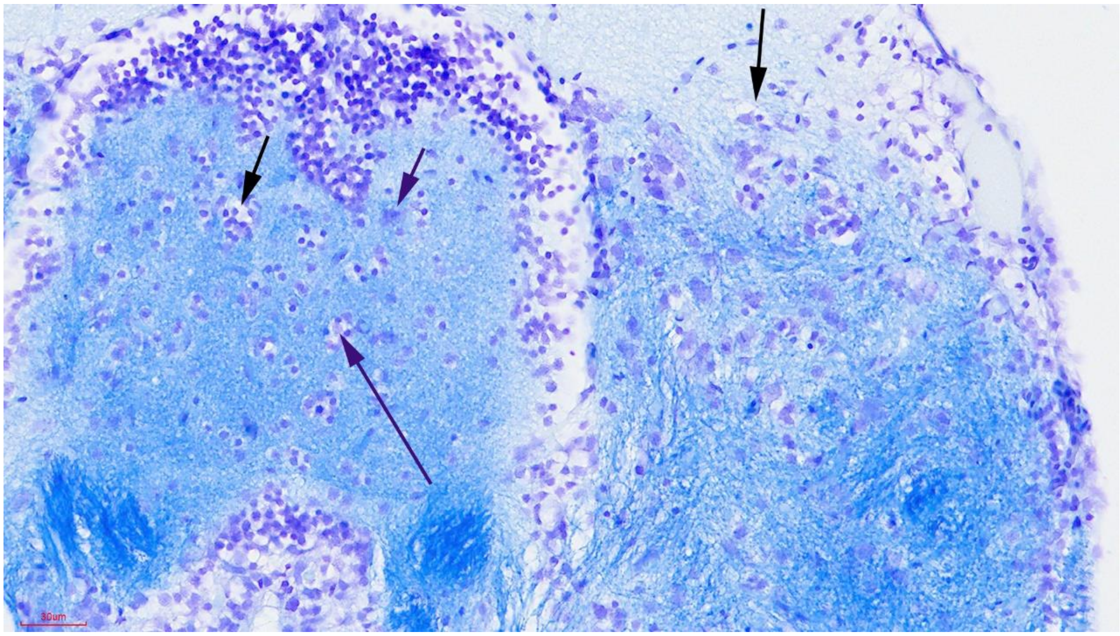

Supplementary Figure 7. Advanced neuroinflammation with reactive glial cells (violet arrows) surrounding degenerating neurons in the medulla oblongata from the DM-IMI-treated group. Severe vacuolation is evident (black arrows), along with increased gliosis (violet arrows). This suggests a chronic neurotoxic response. Staining: Luxol Fast Blue-Cresyl Violet. Magnification: 40x.

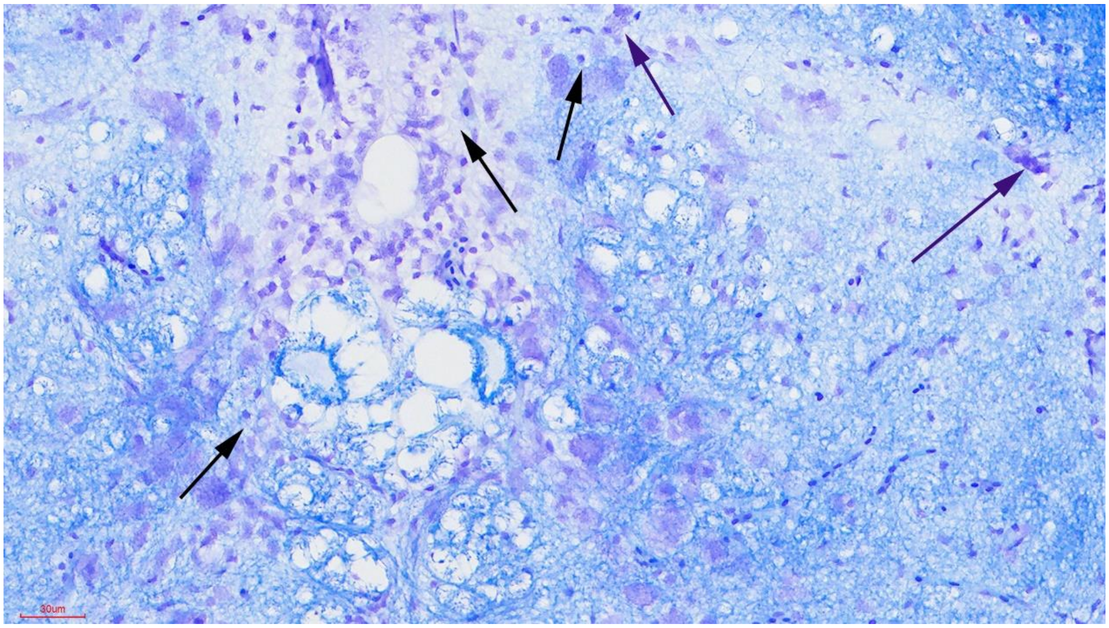

Supplementary Figure 8. The histopathological changes in the medulla oblongata from the DM-IMI-treated group. Neuronal degeneration is observed (violet arrows), with vacuolation (black arrows), disorganized neuropil, and gliosis (violet arrows). Staining: Luxol Fast Blue-Cresyl Violet. Magnification: 40x.
